# Genome-wide identification and comparative analysis of drought-related microRNAs in two maize inbred lines with contrasting drought tolerance by deep sequencing

**DOI:** 10.1371/journal.pone.0219176

**Published:** 2019-07-05

**Authors:** Xuyang Liu, Xiaojing Zhang, Baocheng Sun, Luyang Hao, Cheng Liu, Dengfeng Zhang, Huaijun Tang, Chunhui Li, Yongxiang Li, Yunsu Shi, Xiaoqing Xie, Yanchun Song, Tianyu Wang, Yu Li

**Affiliations:** 1 Institute of Crop Science, Chinese Academy of Agricultural Sciences, Beijing, China; 2 Institute of Grain Crops, Xinjiang Academy of Agricultural Sciences, Urumqi, China; National Taiwan University, TAIWAN

## Abstract

Drought has become one of the most serious abiotic stresses influencing crop production worldwide. Understanding the molecular regulatory networks underlying drought adaption and tolerance in crops is of great importance for future breeding. microRNAs (miRNAs), as important components of post-transcriptional regulation, play crucial roles in drought response and adaptation in plants. Here, we report a miRNome analysis of two maize inbred lines with contrasting levels of drought tolerance under soil drought in the field. Differential expression analysis showed 11 and 34 miRNAs were uniquely responded to drought in H082183 (drought tolerant) and Lv28 (drought sensitive), respectively, in leaves. In roots, 19 and 23 miRNAs uniquely responded to drought in H082183 and Lv28, respectively. Expression analysis of these drought-responsive miRNA-mRNA modules revealed miR164-MYB, miR164-NAC, miR159-MYB, miR156-SPL and miR160-ARF showed a negative regulatory relationship. Further analysis showed that the miR164-MYB and miR164-NAC modules in the tolerant line modulated the stress response in an ABA (abscisic acid)-dependent manner, while the miR156-SPL and miR160-ARF modules in the sensitive line participated in the inhibition of metabolism in drought-exposed leaves. Together, our results provide new insight into not only drought-tolerance-related miRNA regulation networks in maize but also key miRNAs for further characterization and improvement of maize drought tolerance.

## 1 Introduction

To meet the rising demand resulting from population growth and economic development, crop production should maintain a sustainable increase [1]. However, the increasing environmental stresses resulting from global climate change, such as drought, have become major threats to crop production [2]. Maize (*Zea mays* L.) is one of the most important crops for food and feed worldwide [3]. Although maize yield has grown over the past decades, maize has also demonstrated increasing sensitivity to drought [4]. Thus, breeding of maize varieties with drought tolerance is an important alternative approach to relieving the threat; however, it requires a better understanding of the molecular regulatory networks of drought tolerance.

microRNAs (miRNAs), which are endogenous, short, single-stranded non-coding RNAs, play key roles in the stress response and various developmental processes in plants as post-transcriptional regulators [5, 6]. miRNAs suppress the expression of target genes by binding to the partially complementary sequences in messenger RNAs (mRNAs) and then cleaving and degrading mRNA molecules or inhibiting translation with the cooperation of RNA-induced silencing complexes (RISCs) [7]. The target genes of miRNAs can be predicted based on bioinformatic analysis of miRNA-mRNA complementary sequences [8]. And the degradome sequencing also provides supporting evidence of interactions between miRNAs and their target genes [9, 10]. Most target genes of miRNAs in plants are transcription factors (TFs, 65.9%), followed by NB-LRR proteins (nucleotide-binding domain and leucine-rich repeat domain, 6.6%), pathogen proteins (2.2%), lncRNAs (long non-coding RNAs, 1.1%) and other proteins (24.2%) [5].

The genome-wide identification of miRNAs that respond to drought has been reported in plants by using microarrays [11–15] or by RNA sequencing (RNA-Seq) [16–20]. Numerous miRNAs have been reported to respond to drought in different plant species, such as *Arabidopsis* [21], rice (*Oryza sativa* L.) [19], wheat (*Triticum aestivum* L.) [22] and soybean (*Glycine max* L.) [16]. For example, in *Arabidopsis*, miR393, miR397b and miR402 have been shown to be up-regulated while miR319c and miR389a were down-regulated under dehydration stress [23]. In another study, 17 miRNAs, including miR164c, miR319b, and miR1861d, were down-regulated under drought in rice at the seedling stage, while 16 miRNAs (miR166h, miR172d, miR408, etc.) were up-regulated [24].

In the maize genome, 321 mature miRNAs derived from 172 precursors, which could be divided into 29 families, were identified [25, 26]. Many miRNAs from different families have been reported to respond to drought in maize [27]. For example, a maize inbred line with drought tolerance was treated by simulated drought (16% polyethylene glycol, PEG) at the seedling stage and miRNA expression analysis showed that 68 miRNAs responded to drought, with 25 up-regulated and 43 down-regulated miRNAs [15]. Two drought-tolerant and two drought-sensitive inbred lines were treated by simulated drought (16% PEG for 16 and 24 hours), and the results of the miRNA microarray analysis identified 303 differentially expressed miRNAs under drought, with 177 up-regulating and 126 down-regulating [28]. It was also found that eight miR169 members could target seven *NF-YA* (*NUCLEAR FACTOR-Y subunit A*) genes in maize, and they responded to PEG treatment in reverse correlation patterns in leaves and roots [29, 30].

Previous studies showed that the responses of miRNAs to drought stress are highly complex because the expression patterns of the same miRNAs under drought might vary among plant species, tissues, treatment methods or even different genotypes of the same species [27]. For example, miR172 was up-regulated in *Arabidopsis* while it was down-regulated in rice in response to drought [14, 31]. Four miRNAs (miR397a/b, miR398b, miR408-5p and miR528-5p) were down-regulated in drought-tolerant rice varieties but up-regulated in a drought-sensitive variety [19]. With respect to drought treatments, rapid soil drying in limited pots and under simulated drought with PEG were shown to vary significantly from real drought conditions in the field [32]. Hence, different drought treatments may lead to contradictory results pertaining to miRNA regulation patterns. For instance, in maize, miR398 was up-regulated under PEG treatment but down-regulated under soil drought [13, 15]. Therefore, it is of great importance to detect the actual regulatory mechanisms of the miRNome under soil drought conditions in the field from the viewpoint of plant breeders.

To gain a better understanding of the regulatory mechanisms performed by miRNAs to improve drought tolerance, we report a miRNome study using two maize inbred lines with contrasting levels of drought tolerance during soil drought in the field. The aims of this study were to identify the responding miRNAs under soil drought, compare the different regulatory patterns between the drought-tolerant and drought-sensitive lines and investigate the candidate miRNAs with potential for genetic improvement of drought tolerance in maize.

## 2 Materials and methods

### 2.1 Plant materials, field experimental design and leaf relative water content measurement

Two maize inbred lines, i.e., H082183 and Lv28, were used in this study. H082183 was an inbred line with strong drought tolerance that was obtained by double haploid (DH) technology and the seeds for which were provided by Prof. Jiuran Zhao of the Maize Research Center of Beijing Academy of Agricultural and Forestry Sciences. Lv28 was a drought-sensitive inbred line and a representative of the Luda Red Cob heterotic group in China [33]. The field experimental design has been previously described [34]. Briefly, these two materials were planted in the field at Urumqi (Xinjiang, China; 43.98°N, 87.51°E), which features limited rainfall and an enormous evaporation capacity. For each inbred line, two water treatment and six biological replicates were established with two row plots containing 11 plants for each row. The rows were 3 meters long and spaced 0.6 meters apart. All plants were watered by drip irrigation at the germination stage at sowing. Plastic films for uniform germination were removed at stage V4. Plants in the well-watering treatment were then irrigated every week, while watering was withheld from plants in the drought treatment. To evaluate the physiological and molecular responses of the plants to drought, the last fully expanded leaves of both genotypes and treatments were sampled to measure the leaf relative water content (RWC); moreover, the second-to-last fully expanded leaves (10 cm leaf tips) and roots (10 cm root tips) were sampled and frozen in liquid nitrogen immediately and stored at -80°C for small RNA sequencing. Sampling was performed at 11:00 (GMT +8:00; 9:00 local time), and the RWC measurement was performed according to the Barrs method [35].

### 2.2 RNA extraction, library construction and Illumina sequencing

According to the leaf RWC results, the samples at 27 and 46 days after drought (DAD), which corresponded to moderate drought and severe drought, respectively, were chosen for sequencing. The samples at 27 and 46 days after drought under the well-watered condition were also selected as the moderate drought control and severe drought control. For each genotype and treatment, the leaf and root samples from two individual plants with the closest RWC in six replicates were selected as two biological replicates for sequencing. In total, 32 samples of the two genotypes (H082183 and Lv28), two tissues (leaf and root), two treatments (drought and well-watered), two drought levels (moderate and severe drought) and two biological replicates were used for miRNA and mRNA sequencing. The mRNA sequencing results have been published in our previous studies [34] (SRA accession number: SRP102142, SRP119805).

For miRNA sequencing, the total RNA of 32 samples was extracted using TRIzol (Invitrogen, USA) according to the manufacturer’s protocol. The purity of the total RNA was examined using a NanoDrop 2000 spectrophotometer (Thermo Fisher Scientific, USA). The concentration and integrity of the RNA were measured with a Qubit 2.0 fluorometer (Thermo Fisher Scientific, USA) and Bioanalyzer 2100 (Agilent Technologies, USA), respectively. The RNA samples with OD260/280 > 1.8 and OD260/230 > 1.0 and RNA concentration > 250 ng/μL and RIN > 8.0 were selected for the following small RNA library construction. Small RNA sequencing libraries were constructed using the Next Ultra Small RNA Sample Library Prep Kit for Illumina (NEB, USA). Briefly, small RNA molecules were ligated with 3’ and 5’ adapters by T4 RNA Ligase. Subsequently, RNA samples were reverse-transcribed and amplified by PCR. Then, the cDNA was separated by 6% polyacrylamide gel electrophoresis (PAGE), and the short molecules were recovered and purified. The Illumina HiSeq 2500 (Illumina, USA) platform was employed to sequence the 32 small RNA samples at Biomarker Technologies (China).

### 2.3 Alignment and mapping of the small RNA sequence data

The sequence read length was set to 50 nucleotides (nt). Raw reads with low sequencing quality, and with lengths shorter than 18 nt or longer than 30 nt, were eliminated to produce clean data. The clean read data were deposited in the NCBI Sequence Read Archive (SRA, https://www.ncbi.nlm.nih.gov/sra, accession number: SRP119798). To identify known non-coding RNAs and repetitive sequences, clean reads were aligned to ribosome RNA (rRNA) sequences in Silva (http://www.arb-silva.de) [36], transfer RNA (tRNA) sequences in GtRNAdb (http://lowelab.ucsc.edu/GtRNAdb) [37], small nuclear RNA (snRNA) and small nucleolar RNA (snoRNA) sequences in Rfam (http://rfam.xfam.org) [38], and repetitive sequences in Repbase (http://www.girinst.org/repbase) [39], using Bowtie software [40]. The clean reads were also aligned to the maize reference genome (*Zea mays* AGPv3.24, ftp://ftp.ensemblgenomes.org/pub/plants/release-24/fasta/zea_mays) [3] using miRDeep2 [41].

### 2.4 Identification of known and novel miRNAs

miRDeep2 [41] was used to align the clean reads to miRBase release 22 (http://www.mirbase.org) [26] to identify known miRNAs. The identification of novel miRNA in maize was performed according to the 2018 criteria for plant miRNA annotations [42]. First, the reads obtained by small RNA sequencing were mapped to the reference genome by miRDeep2, and the secondary structure of miRNA precursors was predicted by the RNAfold program [43]. The resulting secondary structure was analyzed to verify characteristic miRNAs based on the following criteria: (1) miRNA-miRNA* duplex formed with two-nucleotide 3’ overhangs and a foldback size less than 300 nucleotides; (2) no more than five mismatched positions, only three of which were nucleotides in asymmetric bulges; (3) an miRNA length ≥ 20 and ≤ 24; and (4) novel miRNAs met all criteria in at least three small RNA sequencing libraries.

For the candidate novel miRNAs, a stricter criterion was used to obtain credible prediction results, which stated that the novel miRNAs should contain the target sequence, as confirmed by degradome sequencing. The novel miRNAs that fit all the criteria were also confirmed by stem-loop reverse transcription PCR. Small RNAs of the maize inbred lines B73, H082183 and Lv28 were extracted using the miRcute miRNA isolation kit (Tiangen Biotech, China) and reverse-transcribed to cDNA with stem-loop reverse transcription primers using the miRcute plus miRNA first-strand cDNA synthesis kit (Tiangen Biotech, China). PCR amplification was performed using 2×EasyTaq PCR SuperMix (Transgen Biotech, China). The sequences of primers were listed in (**Table A in S1 File)**.

The pre-miRNA and mature miRNA sequences of maize (*Zea mays*), *Arabidopsis thaliana*, rice and sorghum (*Sorghum bicolor* L.) were downloaded from miRbase release 22 (http://www.mirbase.org) [26] and used in the phylogenetic analysis performed with MEGA5 [44].

### 2.5 microRNA expression analysis

For each miRNA, gene expression was calculated and normalized using the TPM algorithm [45]. The TPM value was used for principal component analysis (PCA) and cluster analysis of the miRNome profiling of each sample. DESeq [46] was used to analyze the differentially expressed genes. To detect the drought-responsive miRNAs of each genotype, differential expression analysis was performed for the same genotype subjected to different watering treatments (drought or well-watered conditions), with four comparison groups, i.e., H082183 under moderate drought *vs*. well-watered control, Lv28 under moderate drought *vs*. well-watered control, H082183 under severe drought *vs*. well-watered control, and Lv28 under severe drought *vs*. well-watered control. The criteria for differential expression were established as |log_2_(fold change)| > 1 and FDR < 0.05.

### 2.6 Prediction and annotation of microRNA target genes

The target genes of miRNA were predicted by TargetFinder software [8] with default parameters. The function and annotation of target genes was analyzed using the Blast to Swiss-Prot (http://www.uniprot.org/) [47], NCBI non-redundant protein sequence (Nr) (ftp://ftp.ncbi.nih.gov/blast/db/) [48] and Pfam databases (http://pfam.xfam.org/) [49]. The drought-responsive genes, obtained by mRNA sequencing in our previous studies [34], were calculated using the same methods with miRNAs, with criteria of |log_2_(fold change)| > 1 and FDR < 0.05 (some genes with |log_2_(fold change)| > 0.6 and FDR < 0.05 were also considered differentially expressed genes).

### 2.7 Identification of co-expressed genes and binding sequence of transcription factor genes

Clustering analysis of mRNA genes was performed to identify the genes co-expressed with miRNA target genes. The *k*-means cluster analysis was performed using R, with *k* = 25 and *n* of the cluster = 20. The co-expressed genes with miRNA target transcription factor genes were used for the binding sequence analysis. The motif sequence scan of *cis*-regulatory elements of co-expressed genes was performed by FIMO [50] using the 2-k sequences in the promoter regions, with a criterion of *P* < 0.0001. The over-representation of *cis*-regulatory elements in promoters was identified by Fisher’s exact test, *P* = *F*(*x*, *y*, *n*, *N*), where *x* was the number of a considered *cis*-elements in an individual promoter, *y* was the total number of *cis*-elements in an individual promoter, *n* was the number of a considered *cis*-element in promoters of all genes in maize, and *N* was the total number of *cis*-elements in promoters in all genes in the maize genome. The significance level of over-representation was defied as *P* < 0.001. Gene Ontology (GO) annotation analysis was performed with the Singular Enrichment Analysis (SEA) tool of agriGO (http://bioinfo.cau.edu.cn/agriGO/) [51], using *Zea mays* AGPv3.30 as the reference. The significance of enriched GO terms was calculated by the Fisher statistical test, and the FDR value were obtained by Bonferroni multi-test adjustment. The threshold of significant enriched GO terms was set to FDR < 0.05.

### 2.8 Degradome sequencing

Two bulked (mixed) samples of H082183 and Lv28 were used for degradome sequencing. The H082183 and Lv28 were planted in the greenhouse and watered every three days. After three weeks of germination, drought treatment was performed by withholding water for 20 days, while the plants of the well-watered treatment were watered normally every three days. The leaves and roots under drought and well-watered treatments of H082183 and Lv28 were sampled and mixed for degradome sequencing. The degradome libraries were constructed following the method of Ma *et al*. [52]. Briefly, the cleaved mRNAs with 5’ monophosphates were ligated to adapters containing a MmeI recognition site. The first strand cDNA was produced using oligo(dT) and PCR. The PCR product was digested with MmeI. After that, a double DNA adapter was ligated to digested PCR product with T4 DNA ligase and amplified by PCR. Then, single-end sequencing of degradome libraries was performed on an Illumina HiSeq 2500 (Illumina, USA). The CleaveLand pipeline [9] was used to identify potentially cleaved targets.

### 2.9 Gene expression under ABA treatment

The H082183 were planted in the greenhouse. After three weeks of germination, the plants were irrigated with 100 μM ABA (abscisic acid). The leaves and roots of H082183 were sampled just before ABA treatment (0 h) and at 6 h, 12 h and 24 h after ABA treatment. Three replicates, which contained three plants in each, were used for subsequent RNA extraction and gene expression analysis.

For the qRT-PCR of mRNA, reverse transcription of mRNA to cDNA was performed using EasyScript One-Step gDNA Removal and cDNA Synthesis SuperMix (Transgen Biotech, China). qRT-PCR was performed using the PowerUp SYBR Green Master Mix (Applied Biosystems, USA) on an ABI QuantStudio3 (Applied Biosystems, USA). Each 20 μL PCR contained 1 μL cDNA, 10 μL PowerUp SYBR Green Master Mix (2X), 0.5 μL forward primer, 0.5 μL universal reverse primer and 8 μL ddH_2_O. The thermal cycling parameters were as follows: 50°C for 2 minutes, 95°C for 2 minutes, 40 cycles of denaturing at 95°C for 15 seconds and annealing extension at 60°C for 1 minute, followed by melting curve analysis. The maize *GAPDH* gene was used as an internal reference. The expression level was calculated by 2^-ΔΔCt^ method [53]. Each sample was also performed with three replicates. The sequences of the primers used in the qRT-PCR are listed in (**Table A in S1 File)**.

### 2.10 Quantitative real-time RT-PCR of miRNAs

To confirm the miRNA expression obtained by sequencing, quantitative real-time RT-PCR (qRT-PCR) of five miRNAs (miR166e, miR172b-3p, miR319b-3p, miR408b-3p and miR528a-5p) in leaves and five miRNAs (miR1432-5p, miR159b-3p, miR172b-3p, miR398a-5p and miR399j-5p) in roots of H082183 and Lv28 under drought was performed. The small RNAs of 16 samples under drought were extracted using the miRcute miRNA Isolation Kit (Tiangen Biotech, China). The first-strand cDNA fragments were synthesized using the miRcute Plus miRNA First-Strand cDNA Synthesis Kit (Tiangen Biotech, China). Briefly, a poly(A) tail was added to miRNAs by *E*. *coli* Poly(A) Polymerase. Then, Oligo(dT)-Universal Tag primers were used to generate reverse-transcript cDNA of the miRNAs. qRT-PCR was performed using the miRcute miRNA qPCR Detection Kit (SYBR Green) (Tiangen Biotech, China) on an ABI7500 (Applied Biosystems, USA). Each 20 μL PCR contained 1μL cDNA, 10 μL 2×miRcute miRNA Premix (with SYBR&ROX), 0.4 μL forward primer, 0.4 μL universal reverse primer and 8.2 μL ddH_2_O. The thermal cycling parameters were as follows: 95°C for 2 minutes, 40 cycles of denaturing at 95°C for 20 seconds and annealing extension at 60°C for 34 seconds, followed by melting curve analysis. The maize *U6* gene was used as an internal reference. The expression level was calculated by 2^-ΔΔCt^ method [53]. Each sample was performed with three replicates. The expression levels of miRNAs in H082183 and Lv28 under moderate drought or severe drought were transformed to log_2_(H082183/Lv28) for comparison with the sequencing results.

## 3 Results

### 3.1 Physiological response of two maize inbred lines to drought stress in the field

**Fig 1:**
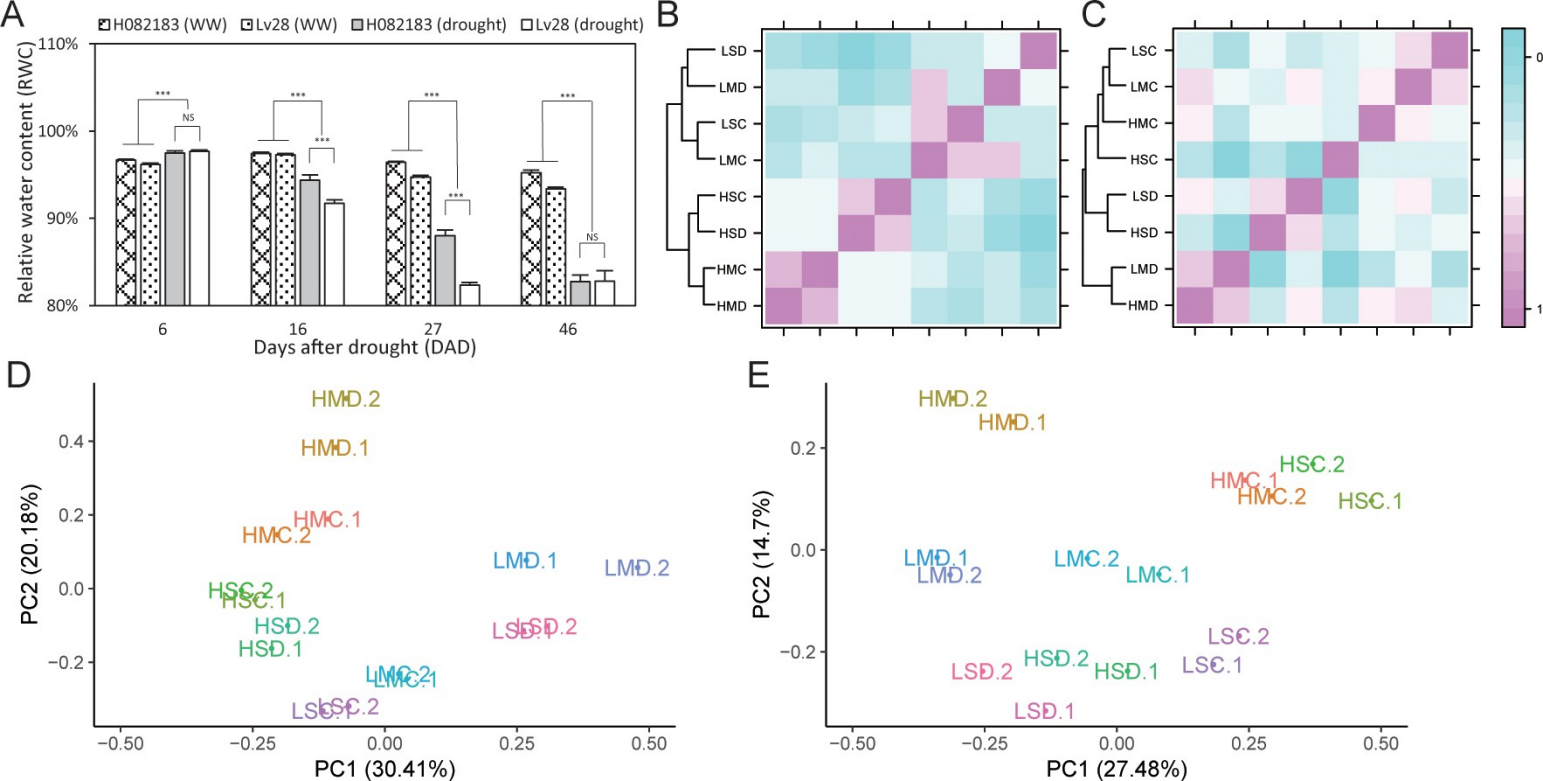
The change in leaf relative water contents under drought and the sample relationship based on the miRNome results. (A) The leaf relative water contents (RWCs) of H082183 and Lv28 under drought and well-watered treatments. **: *P* < 0.01; *: *P* < 0.05; NS: *P* > 0.05. (B) Cluster analysis of miRNome of leaf samples. (C) Cluster analysis of miRNome of root samples. (D) Principal component analysis (PCA) of leaf samples. (E) PCA of root samples. HMD: H082183 under moderate drought, HSD: H082183 under severe drought, LMD: Lv28 under moderate drought, LSD: Lv28 under severe drought. HMC: H082183 under moderate drought control, HSC: H082183 under severe drought control, LMC: Lv28 under moderate drought control, LSC: Lv28 under severe drought control.

Two maize inbred lines, Lv28 and H082183, were grown, and leaf relative water contents (RWCs) were tested under well-watered and soil drought conditions in the field. The results showed that the leaf RWCs of both genotypes under drought were significantly (*P* < 0.001) lower than those of their well-watered controls at 27 and 46 days after drought (**Fig 1A**). At 27 days after drought, the leaf RWCs of H082183 and Lv28 was 89.30% and 84.31% under drought, respectively, and showed significant difference (*P* < 0.001). However, the leaf RWCs of both H082183 and Lv28 decreased to approximately 82%, and showed no significant difference at 46 days after drought.

### 3.2 Sequencing and alignment

To understand drought-responsive and tolerance-related miRNAs in maize, we selected the leaf and root samples of both genotypes at 27 and 46 DAD, representative of moderate and severe drought, for sequencing. In addition to the well-watered controls of moderate drought and severe drought, small RNA libraries of 32 samples were sequenced. In total, 743.8 million raw short reads were obtained, with 18.6 million raw reads per sample on average (**Table B in S1 File**). Reads of low quality and substandard sequence length (fewer than 18 nucleotides or greater than 30 nucleotides) were removed. Therefore, a total of 533.6 million clean reads were acquired, with a mean of 13.3 million clean reads per sample. The Q30 scores of all samples ranged from 94.54% to 99.13%, with an average of 97.32%, indicating that the sequence data were of reliable quality. The clean reads were aligned to the maize reference genome, and the percentage of mapped reads in each library’s clean reads ranged from 13.26% to 29.60%, with a mean value of 18.37% (**Table C in S1 File**). The clean reads were mapped to different small RNA databases using Bowtie to differentiate the types of small RNAs. The most abundant small RNA was rRNA, with an average percentage of 43.73%, followed by tRNA (7.77%), repeated sequences (1.60%) and snoRNA (0.17%) (**Table D in S1 File**). The unannotated reads were aligned to miRBase (release 22) to identify the known miRNAs in maize. It was found that 278 and 275 known miRNA genes were expressed in leaf and root samples, respectively (**Table E in S1 File**). Those known miRNAs could be classified into 28 miRNA families (**Fig A in S1 File**). In addition, the length distribution of the expressed known miRNAs was highly enriched in 21, 20 and 22 nucleotides (nt) (**Fig B in S1 File**).

Two bulked (mixed) samples of H082183 and Lv28 were used for degradome sequencing to confirm the predicted target genes of the miRNAs. A total of 30.6 and 25.4 million reads were obtained by degradome sequencing in H082183 and Lv28, respectively (**Table F in S1 File**). In addition, 99.59% and 99.55% of the degradome sequencing reads could be mapped to the maize reference genome (*Zea mays* AGPv3.24), and covered 36417 and 37862 transcripts in H082183 and Lv28, respectively.

### 3.3 Novel miRNAs in maize

**Fig 2:**
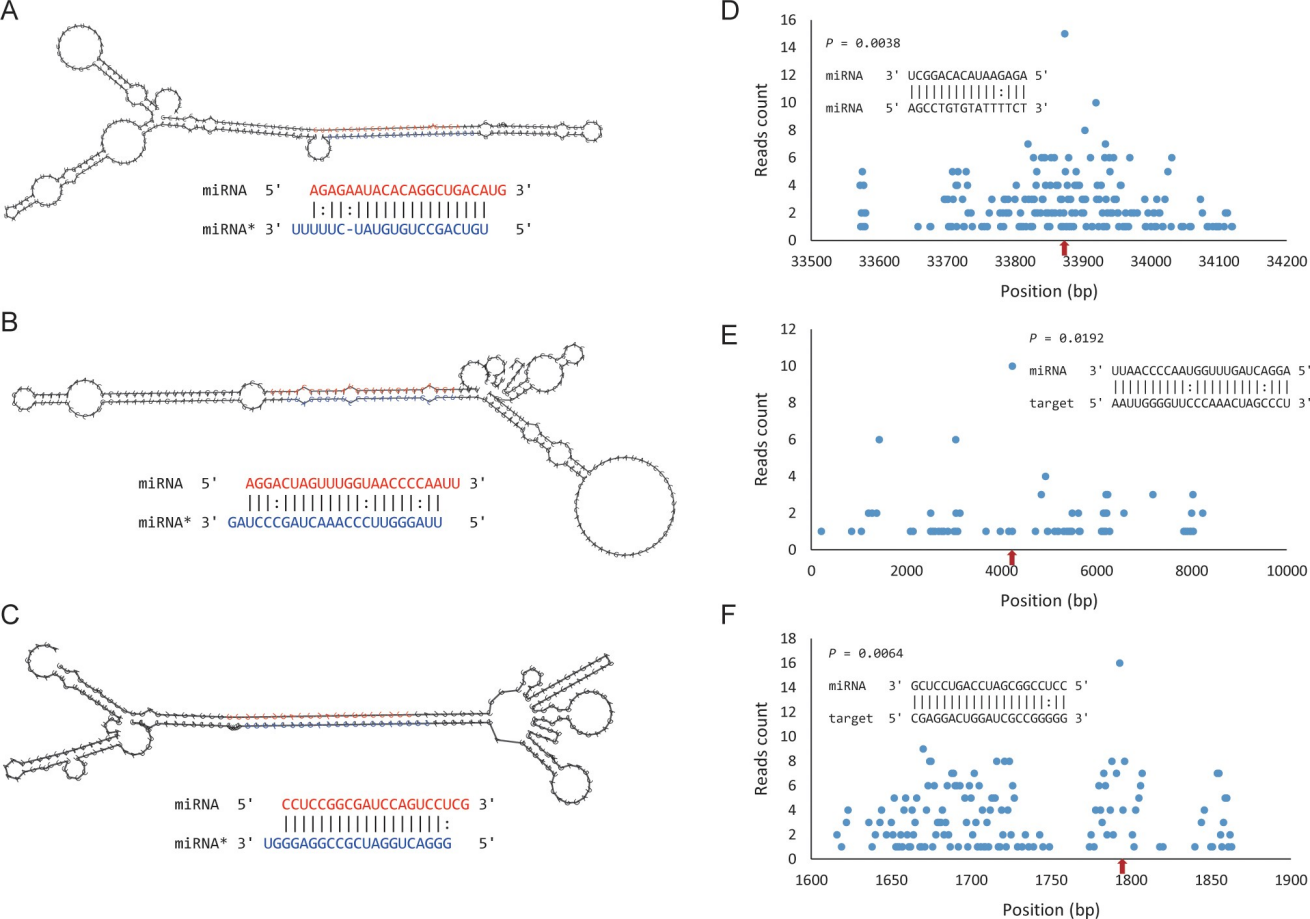
The second structure and target sequence of predicted novel miRNAs. The stem-loop structure and miRNA-miRNA* duplex of Novel_2_38 (A), Novel_10_88 (B) and Novel_10_147 (C). The target sequence and cleavage read count by degradome sequencing of Novel_2_38-GRMZM2G308707 (D), Novel_10_88-AC190628.4_FG007 (E) and Novel_10_147-GRMZM2G064212 (F).

A total of 34 novel miRNAs were identified based on the 2018 criteria for plant miRNA annotations [42] (**Table G in S1 File**). The lengths of novel miRNAs were enriched in 21 and 24 nt. Six of the novel miRNAs were conserved with other plant species (**Fig C in S1 File**). Three novel miRNAs (named Novel_2_38, Novel_10_88 and Novel_10_147 according to their positions on chromosomes) were confirmed by degradome sequencing (**Fig 2**). The Novel_2_38, Novel_10_88 and Novel_10_147 miRNAs could cleave the 33873, 4231, and 1783 site of GRMZM2G308707, AC190628.4_FG007, and GRMZM2G064212, respectively. The phylogenetic results showed that the novel miRNAs Novel_2_38, Novel_10_88 and Novel_10_147 were close to osa-miR1905, osa-miR5534 and sbi-miR6229, respectively (**Fig D in S1 File**). However, the mature sequences of these three novel miRNAs did not have conservative seed regions with their relative miRNAs in other species, indicating these novel miRNAs were not annotated by homologies in plants. In addition, the Novel_2_38, Novel_10_88 and Novel_10_147 were confirmed by stem-loop reverse transcription and PCR amplification in different maize lines.

### 3.4 Expression profiles of miRNAs

The miRNA expression profiles of each of the two biological replicates samples were all significantly correlated (*r* > 0.95, *P* < 0.01) (**Fig E in S1 File**). Principal component analysis (PCA) showed the samples were closest to their corresponding biological samples in both leaves and roots (**Fig 1D and 1E**). The clustering of different leaf samples showed that the samples were first divided into two groups by genotypes and then separated by treatment (**Fig 1B**), while the root samples were initially clustered by drought treatments and subsequently by different genotypes (**Fig 1C**).

### 3.5 Drought-responsive miRNAs

**Fig 3:**
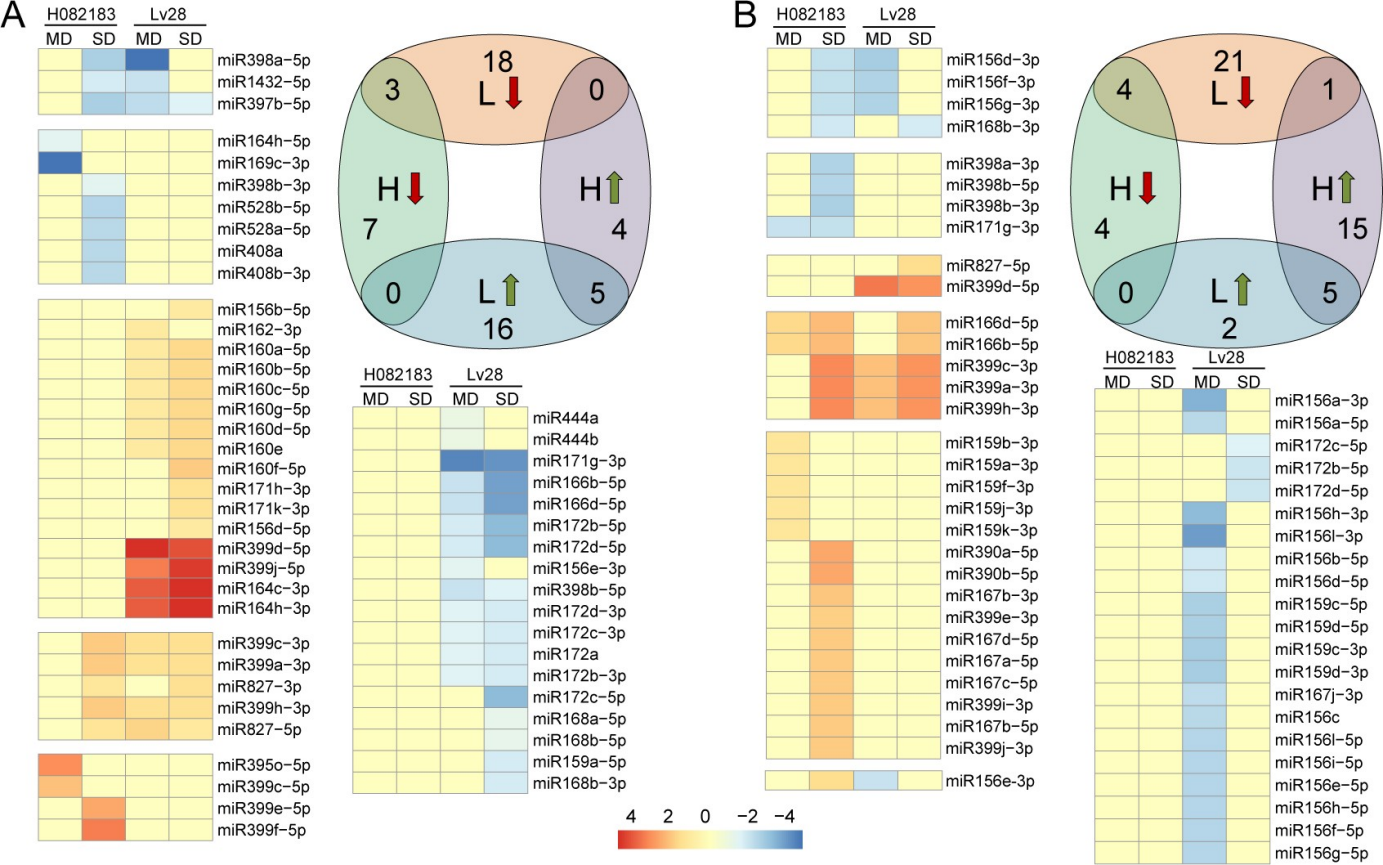
Drought-responsive miRNAs in leaves and roots. (A) The drought-responsive miRNAs in leaves. (B) The drought-responsive miRNAs in roots. The arrows in the Venn plot indicate down- or up-regulation of miRNAs. MD: moderate drought, SD: severe drought.

The drought-responsive miRNAs in HMD, LMD, HSD and LSD were acquired by comparing the expression profiles of HMC-HMD, LMC-LMD, HSC-HSD and LSC-LSD. In leaves, there were three (miR397, miR398, miR1432) and five miRNAs (miR399s and miR827s) that were commonly down- and up-regulated under drought in both H082183 and Lv28, respectively (**Fig 3A**), while seven (miR164, miR169, miR398, miR528s and miR408s) and four miRNAs (miR395 and miR399s) were exclusively down- and up-regulated in response to drought, respectively, in leaves of H082183. Additionally, 18 (miR156s, miR160s, miR162, miR164s, miR171s and miR399s) and 16 (miR156, miR159, miR166s, miR168s, miR171, miR172s and miR444s) miRNAs were down- and up-regulated, respectively, under drought in leaves of Lv28. In roots, four (miR156s and miR168) and five miRNAs (miR166s and miR399s) were commonly down- and up-regulated under drought in both H082183 and Lv28, respectively (**Fig 3B**). In addition, four (miR398s and miR171) and fifteen (miR159s, miR167s, miR390s and miR399s) miRNAs were exclusively down- and up-regulated in roots of H082183, and twenty-one (miR156s, miR167 and miR172s) and two miRNAs (miR399 and miR827) were uniquely down- and up-regulated under drought in roots of Lv28, respectively. Interestingly, one member of the miR156 family (miR156e-3p) was up-regulated in roots of H082183 but down-regulated in roots of Lv28. However, no predicted novel miRNAs showed a response to drought in leaves or roots.

### 3.6 Target genes of drought-responsive miRNAs

**Fig 4:**
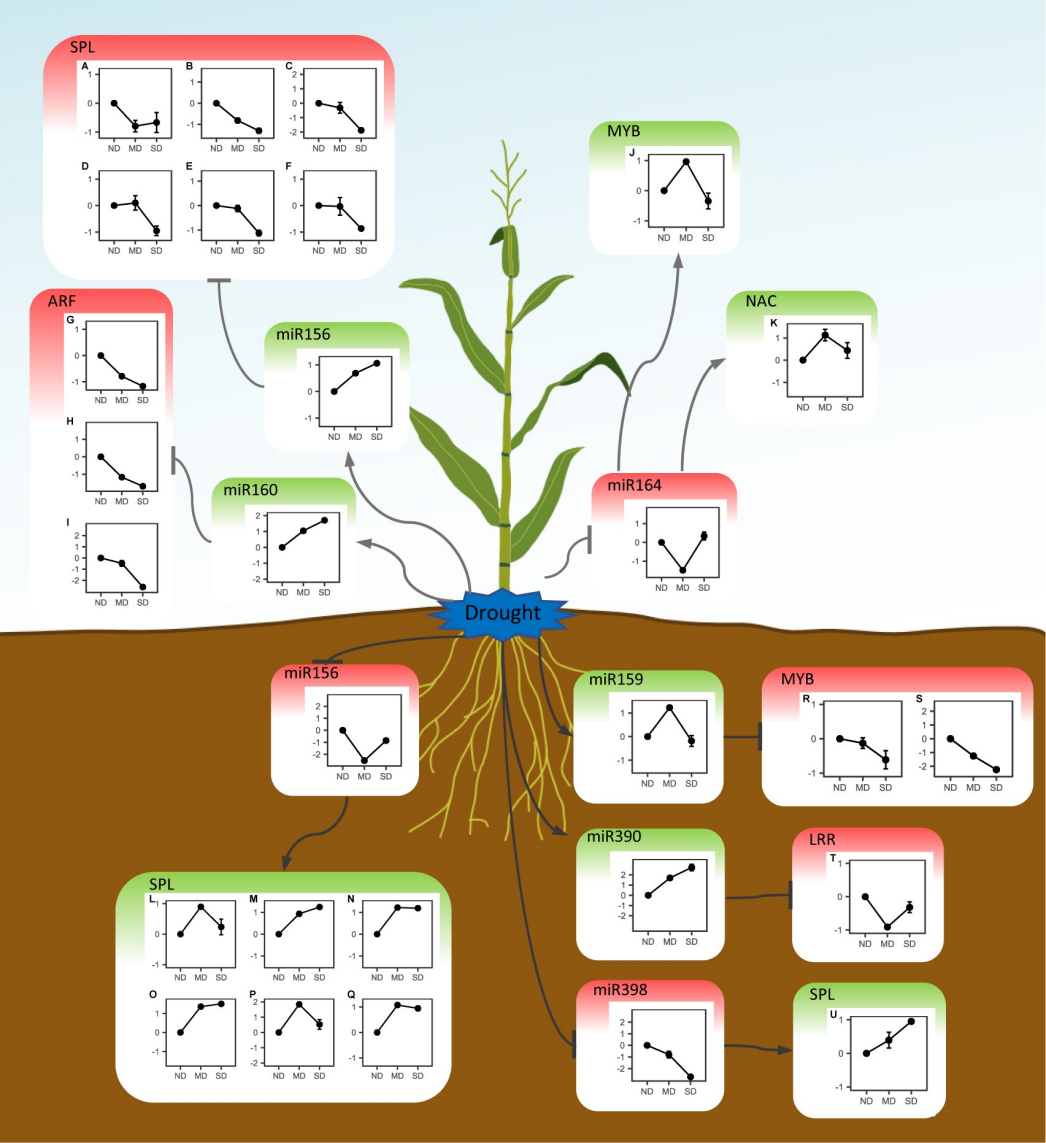
Regulation networks of drought-responsive miRNAs and their target mRNAs. The left and right panel include the miRNA-mRNA expression in Lv28 and H082183 respectively. (A) GRMZM2G065451, (B) GRMZM2G113779, (C) GRMZM2G414805, (D) GRMZM2G126018, (E) GRMZM2G371033, (F) GRMZM2G067624, (G) AC207656.3_FGT002, (H) GRMZM2G390641, (I) GRMZM2G159399, (J) GRMZM2G096358, (K) GRMZM2G114850, (L) GRMZM2G113779, (M) GRMZM2G160917, (N) GRMZM5G806833, (O) GRMZM2G307588, (P) GRMZM2G460544, (Q) GRMZM2G061734, (R) GRMZM2G004090, (S) GRMZM2G028054, (T) GRMZM2G304745, (U) GRMZM2G103812. The y-axis shows the log_2_(fold change) of miRNAs or mRNAs under drought. The x-axis presents the water treatment conditions. ND: well-watered treatment, MD: moderate drought, SD: severe drought.

Among all the miRNAs expressed in this study, 211 were predicted to target 262 mRNAs in maize leaves or roots (**Table H in S1 File**). The expression analysis revealed several target miRNA-mRNA modules that had negative expression patterns. In the leaves of H082183, miR164 was down-regulated under moderate drought, while its target genes GRMZM2G096358 (MYB) and GRMZM2G114850 (NAC) were up-regulated under MD (**Fig 4**). In the roots of H082183, three miRNA-mRNA pairs were negatively regulated under drought, including miR159 and two MYB genes (GRMZM2G004090 and GRMZM2G028054), miR390 and one leucine-rich repeat protein (LRR) gene (GRMZM2G304745), miR398 and one selenium-binding protein (SBP) gene (GRMZM2G103812). In the drought-sensitive line Lv28, miR160 was up-regulated under drought, while its target auxin response factor (ARF) genes AC207656.3_FGT002, GRMZM2G390641 and GRMZM2G159399 were down-regulated under drought. The miR156 displayed the opposite regulation patterns in leaves and roots of Lv28. The up-regulation of miR156 negatively modulated the squamosa promoter binding like protein (SPL) genes GRMZM2G065451, GRMZM2G113779, GRMZM2G414805, GRMZM2G126018, GRMZM2G371033 and GRMZM2G067624. However, miR156 was down-regulated in the roots of Lv28, and the target SPL genes (GRMZM2G113779, GRMZM2G160917, GRMZM5G806833, GRMZM2G307588, GRMZM2G460544 and GRMZM2G061734) were up-regulated under drought. These miRNA-mRNA modules were confirmed by degradome sequencing (**Fig F in S1 File**).

### 3.7 Regulatory networks of drought-responsive miRNAs

Transcription factor (TF) genes regulate gene expression by binding to the *cis*-element in promoter regions. To explore the regulatory networks of the miRNA-TF pairs, a clustering analysis was performed to detect the genes co-expressed with the target mRNA genes (**Fig G in S1 File**). For example, 341 genes were co-expressed with the miR164-targeted MYB (GRMZM2G096358) and NAC (GRMZM2G114850) genes in leaves of H082183. Additionally, 364 genes were co-expressed with the miR156-targeted MYB (GRMZM2G028054) gene in roots of H082183. Furthermore, the co-expressed genes with MYB, NAC, ARF or SPL binding motifs in their promoter regions were selected for GO enrichment analysis. The results revealed that the putative modulated genes of the miR164-MYB and miR164-NAC modules mostly involved the GO terms “response to stimulus”, “response to stress” or “response to water deprivation” in the leaves of H082183 (**Table 1**). In contrast, the miR156-SPL and miR160-ARF modules in the leaves of Lv28 were both related to “photosynthesis”. In roots, the miR159-MYB modules of H082183 were involved in “membrane”, “plasma membrane” and “response to stimulus”. The miR156-SPL modules were involved in “response to stress”, “response to stimulus” and “response to abiotic stimulus”. These results suggested that the miR164-MYB and miR164-NAC modules in the tolerant line H082183 were involved in the regulation of stress-responsive genes, while the miR156-SPL and miR160-ARF modules in the sensitive line Lv28 participated in inhibition of metabolism and development in leaves. Further analysis revealed 12 and 8 genes that were co-expressed with miR164-MYB and miR164-NAC and that had significantly over-represented MYB and NAC binding motif sequences in the 2-k promoter regions, respectively (**Fig H in S1 File**).

**Table 1:** GO enrichment of genes co-expressed with miRNA target transcription factors.

Tissue	Genotype	TF gene ^a^	GO accession	Type ^b^	Term	Gene No.	FDR
Leaf	H082183	miR164-MYB	GO:0050896	P	Response to stimulus	56	0.00033
			GO:0050794	P	Regulation of cellular process	48	0.00048
			GO:0006950	P	Response to stress	40	0.00048
			GO:0009628	P	Response to abiotic stimulus	28	0.0019
			GO:0009414	P	Response to water deprivation	10	0.0054
		miR164-NAC	GO:0065007	P	Biological regulation	38	8.40×10^−05^
			GO:0009628	P	Response to abiotic stimulus	19	0.00086
			GO:0009266	P	Response to temperature stimulus	10	0.0031
			GO:0006950	P	Response to stress	23	0.0056
			GO:0009408	P	Response to heat	6	0.0056
	Lv28	miR160-ARF	GO:0015979	P	Photosynthesis	18	0.0021
			GO:0070008	F	Serine-type exopeptidase activity	8	0.03
			GO:0004180	F	Carboxypeptidase activity	9	0.03
			GO:0004185	F	Serine-type carboxypeptidase activity	8	0.03
			GO:0004674	F	Protein serine/threonine kinase activity	46	0.03
		miR156-SPL	GO:0009579	C	Thylakoid	29	0.0046
			GO:0009765	P	Photosynthesis, light harvesting	8	0.0062
			GO:0015979	P	Photosynthesis	18	0.01
			GO:0019684	P	Photosynthesis, light reaction	13	0.013
			GO:0031976	C	Plastid thylakoid	21	0.026
Root	H082183	miR159-MYB	GO:0016020	C	Membrane	361	3.2×10^−07^
			GO:0005886	C	Plasma membrane	167	0.000076
			GO:0050896	P	Response to stimulus	231	0.0008
			GO:0051179	P	Localization	157	0.018
			GO:0010038	P	Response to metal ion	49	0.024
	Lv28	miR156-SPL	GO:0006950	P	Response to stress	44	0.0025
			GO:0050896	P	Response to stimulus	58	0.0073
			GO:0009628	P	Response to abiotic stimulus	28	0.044

^a^ The miRNA-target transcription genes

^b^ C: Cell component, F: Molecular function, P: Biological process.

**Fig 5:**
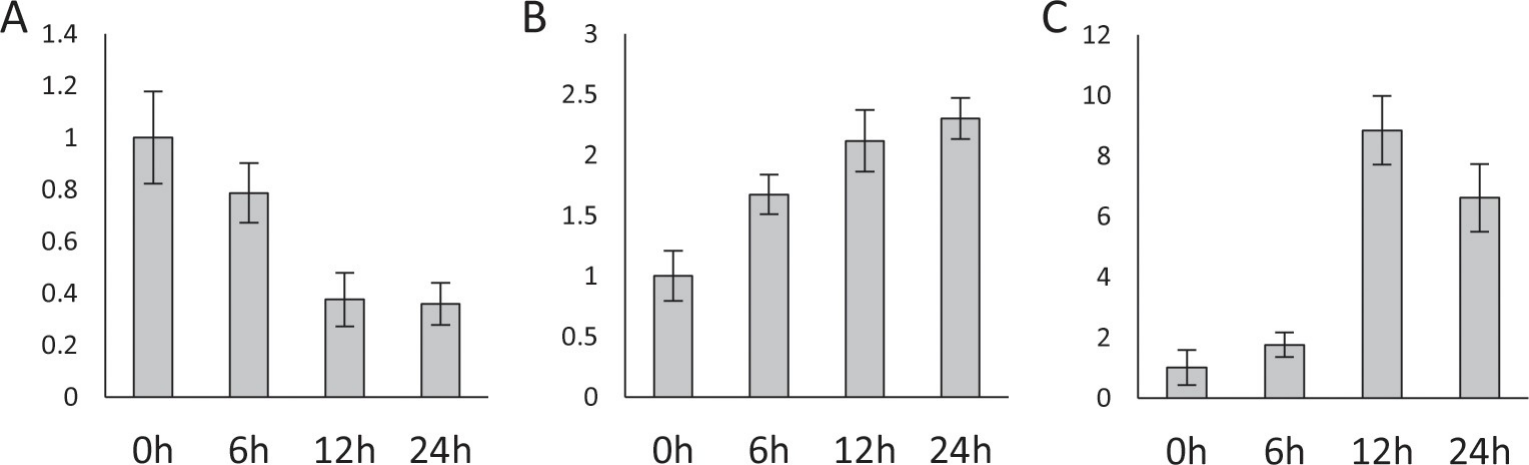
The expression of miR164 and target genes in leaves under ABA treatment. (A) miR164. (B) GRMZM2G114850 (NAC). (C) GRMZM2G096358 (MYB).

The MYB and NAC transcription factors are known to regulate the drought stress response *via* abscisic acid (ABA) signaling pathways [54, 55]. Thus, the expression levels of the miR164-MYB, miR164-NAC and miR159-MYB modules were analyzed under ABA treatment in H082183. In leaves, miR164 was down-regulated, but the target genes GRMZM2G096358 (MYB) and GRMZM2G114850 (NAC) were up-regulated under ABA treatment (**Fig 5**). These results were consistent with the expression patterns of miR164-MYB and miR164-NAC modules under drought (**Fig 4**). However, the negative regulatory relationship of miR159-MYB was lost in roots of H082183 under ABA treatment, which were all down-regulated (**Fig I in S1 File**).

### 3.8 Confirmation of miRNA expression by qRT-PCR

A total of five miRNAs in leaves and five miRNAs in roots were selected for qRT-PCR to confirm the expression of miRNAs obtained by sequencing. The relative expression of miRNAs under drought were calculated as the log_2_(fold change) for comparison with the sequencing results. Correlation analysis showed that the gene expression changes between the two genotypes generated by qRT-PCR and sequencing were significantly correlated (*r* = 0.935, *P* = 3.397×10^−10^) (**Fig J in S1 File**).

## 4 Discussion

### 4.1 Drought-responsive miRNAs in plants

miRNAs, as post-transcriptional regulators, play significant roles in the stress responses of plants. Therefore, a few studies involving the genome-wide identification of drought-responsive miRNAs in various plants have been reported, including *Arabidopsis* [21], rice (*Oryza sativa* L.) [14, 19], and wheat (*Triticum aestivum* L.) [22]. In this study, many miRNAs were detected to respond to drought in leaves and roots. The drought-responsive miRNAs in different plant species were thus compared (**Table I in S1 File**).

In the leaves of maize, miR156, miR160, miR162, miR164, miR171, miR395, miR399 and miR827 were up-regulated under drought, while miR159, miR164, miR166, miR168, miR169, miR171, miR172, miR397, miR398, miR408, miR444, miR528 and miR1432 were down-regulated. Similarly, miR160, miR162, miR395, miR827 were also up-regulated, while miR166, miR172, miR397, miR827 and miR1432 were down-regulated in *Arabidopsis*, rice or wheat [14, 19, 21, 22]. However, certain miRNA families showed different regulatory patterns in plants, i.e., miR159, miR168, miR169, miR398, miR399, miR408, and miR528. For instance, miR159 was down-regulated in maize but up-regulated in rice and wheat under drought [14, 22]. miR168 was downregulated in rice and maize but up-regulated in *Arabidopsis* under drought [14, 21]. miR399 was up-regulated in maize and wheat but down-regulated in rice under drought [19, 22]. In addition, the members of three miRNA families (miR156, miR164 and miR171) showed different regulatory patterns under drought in maize, while miR156 and miR171 also displayed opposite regulations in two individual studies of rice under drought [14, 19].

In roots of maize, miR156, miR159, miR166, miR167, miR390, miR399 and miR827 were up-regulated, while miR156, miR159, miR167, miR168, miR171, miR172 and miR398 were down-regulated, under drought. However, when compared with other plants, only miR398 was conservatively down-regulated in the roots of maize and wheat under drought [22]. miR172 was down-regulated in the roots of maize and leaves of rice and wheat, while it was up-regulated in the roots of wheat under drought [14, 22]. miR399 was up-regulated in the roots of maize and leaves of wheat, but down-regulated in roots of wheat [19, 22]. Furthermore, the members of three miRNA families (miR156, miR159 and miR167) showed altered regulation in maize roots, while miR156 displayed opposite regulations in rice, and miR159 showed a contrasting response between leaves and roots of wheat under drought [14, 19, 22].

### 4.2 Comparison of miRNome generated under field drought conditions and other drought treatment methods

In maize, the genome-wide identification of drought-responsive miRNAs has been reported under simulated drought treatment (PEG) [15, 28] or by withholding water in limited pots in a greenhouse [13, 55]. In this study, drought-induced miRNome using different drought treatment methods were compared, and the regulatory patterns of miRNAs in response to the different drought treatment methods showed great diversity (**Table J in S1 File**). For example, miR159 and miR166 were down-regulated in maize leaves under soil drought in the field or in a greenhouse pot but up-regulated under PEG treatment [13, 28, 55]. miR160 was up-regulated in maize leaves under drought in the field but down-regulated under rapid drought in a limited pot [13]. miR156 was down-regulated under moderate drought but up-regulated under severe drought in the field; however, miR156 was up-regulated and down-regulated at 16 and 24 h after PEG treatment, respectively [15, 28]. miR399 was up-regulated in both leaves and roots under drought in the field but down-regulated under PEG treatment [15]. This finding indicated an inconformity between the effects of soil drought and drought simulated by PEG (dehydration stress) on the expression of mRNAs. Therefore, it is necessary to identify the drought-induced miRNome or transcriptome in the field to determine the actual expression regulatory networks.

### 4.3 miR164 contributed to drought tolerance in leaves of the tolerant line H082183

Transcript regulation at the gene expression level is a major part of the stress responses and has been reported to play a role in the stress tolerance of plants [56]. It is notable that, in plants, the target genes of miRNAs mostly encode transcription factors that play important roles in modulating gene expression at the transcriptomic level [5]. The interaction of miRNAs and transcription factors could regulate the drought stress response and tolerance via different signaling and physiological pathways, such as the ABA (abscisic acid) response, auxin signaling, and osmotic production and antioxidant production [57].

In the present study, the miRNome results suggested the putative role of the miR164-MYB and miR164-NAC modules in stress response regulatory pathways in leaves, and their expression was regulated by ABA. The miR164-NAC modules have been reported related to drought tolerance in rice. For example, Fang *et al*. found that the expression of six NAC genes, which were conservatively targeted by miR164 (*OsmiRNA* targeted *NAC1-6*, *OMTN1-6*), were significantly reduced in leaves under drought. The over-expression transgenic plants of *OMTN* genes showed increased sensitivity to drought stress, which displayed earlier leaf rolling and wilting [58]. In addition, miR164 could target *ZmNAC1* and negatively regulate its expression, and over-expression of *ZmNAC1* contributed to the increased lateral root growth in maize [59]. In wheat, the interaction between miR164 and *TaNAC21/22* was confirmed, and this module could regulate the resistance to stripe rust [60].

Furthermore, the NAC and MYB transcription factor genes played important roles in signal transduction of the drought stress response in plants. Over-expression transgenic *Arabidopsis* of a sorghum NAC gene *SbSNAC1* resulted in improved drought tolerance with a significantly higher survival rate than wild type plants under drought [61]. The expression of *ZmSNAC1* was induced by dehydration treatment, and over-expression of *ZmSNAC1* in *Arabidopsis* enhances tolerance to dehydration stress [62]. The expression of *ZmNAC111* was significantly associated with drought tolerance in maize at the seedling stage [63]. In rice, the expression of *OsMYB3R*-2 was induced by drought, salt and cold stresses, whose over-expression of transgenic *Arabidopsis* led to increased tolerance to drought and salt stresses [64]. The wheat MYB gene *TaMYBsm1* showed functions in improving drought tolerance, the over-expression of which in transgenic *Arabidopsis* led to higher germination rates under drought stress [65]. Additionally, both MYB and NAC TFs were participated in the ABA signal pathway [66]. For instance, the overexpression of *TaNAC29* enhanced the expression of some core genes of the ABA signal pathway, such as *ABI5*, *SAG13* and *SAG113* [67]. And the overexpression of *TaMYB3R1* increased the expression of *RD29A*, *RD29B* and *ABF3*, which were also involved in the ABA signal pathway [68].

### 4.4 miR156 inhibited metabolism and development in leaf but promoted it in root under drought in the sensitive line Lv28

The modules of miR156 and SBL TFs have been shown to regulate many important traits in plants. For example, the OsmiR156 and *OsSPL14* module controlled the ideal architecture in rice, and mutation of the OsmiR156 target site in *OsSPL14* reduced the tiller number and enhanced the grain yield [69]. The SPL TFs have also been shown to regulate grain size, grain quality, panicle branch, and plant height in rice [70, 71]. In maize, miR156 could regulate two SPL TFs, *tga1* and *tsh4*. *tga1* (*teosinte glume architecture1*) has been determined to be a key gene in the domestication of maize from teosinte [72], and the module of miR156 and *tga1* has been shown to regulate the phase transition, tillering and inflorescence architecture [73]. The SBL TF gene *tsh4* (*tasselsheath4*) has been shown to play an essential role in meristem boundary establishment [74]. In *Arabidopsis*, miR156 was up-regulated under drought, and its overexpression resulted in a significantly higher survival rate of transgenic plants under drought stress [75]. Moreover, the miR160-ARF module also plays important role in development in plants. For example, in soybean, overexpression of miR160 in roots results in the hypersensitivity to auxin and cytokinin, resulting in an inhibition of development of the symbiotic nodule [76]. It is interesting that, in our study, the expression of miR156 was induced in leaf bur decreased in root under drought in the drought-sensitive line Lv28, which caused an own-regulation of targeted SPL genes in leaf and up-regulation of the genes in root under drought (**Fig 4**). Additionally, the putative down-stream genes regulated by miR156-SPL modules of Lv28, which showed co-expression with miR156-SPL genes and had squamosal binding sequences in their promoter region, were enriched in photosynthetic carbon metabolism in leaves, while that genes were enriched in stress response function in roots. These results suggested that inhibition of development in shoots and enhancement of development or activation of stress response signal transduction in roots modulated by the miR156-SPL module are strategies for drought stress adaption in maize.

### 4.5 miRNAs with potential in drought tolerance improvement

Some drought responsive miRNAs have shown potentials in drought tolerance improvement. For instance, the deletion of miR169a by CRISPR/Cas9 could enhance drought tolerance in *Arabidopsis*, whose transgenic plants showed a high survival rate under drought [77]. The overexpression of *OsmiR393* down-regulated *OsTIR1* and *OsAFB2* and led to higher sensitivity to drought and salt than the wild type in rice [78]. miR398 has been shown responsive to drought in *Arabidopsis*, rice, pea (*Pisum sativum* L.), and *Medicago truncatula* [79–81]. miR398 was also considered a key miRNA involved in drought tolerance in rice, as it has showed opposite responsive patterns between the tolerant and sensitive cultivars under drought [19]. miR398 conservatively targeted *CSD1* and *CSD2* encoding copper/zinc superoxide dismutases (Cu/Zn-SODs), *COX5b* encoding a subunit of mitochondrial cytochrome c oxidase and *CCS1* encoding the copper chaperone for SOD [82]. The interaction of miR398 and its target genes was also involved in responses to other abiotic stresses, such as heat, salt and oxidative stress [83–85]. In the present study, miR398b was specifically down-regulated in the leaves and roots of H082183 under drought, implying that the miRNA might also be used in stress tolerance improvement. In addition, the miR160-ARFs module could regulate leaf development *via* the auxin signaling pathways, and miR165/166-*HD-ZIP IIIs* module could confer drought tolerance through the ABA signaling pathways in *Arabidopsis* [86]. The double mutant plants of miR160 and miR165/166 showed defects in drought tolerance and leaf development [86]. Our results revealed that the miR160-ARF module was responsive to drought in the leaves of sensitive line Lv28. In *Arabidopsis*, the double mutant of miR159a/b had larger and longer roots than the wild type, and the expressions of target genes *MYB33*, *MYB65* and *MYB101* were increased [87]. In the present study, the miR159-MYB modules showed responsive to drought in roots of H082183. Thus, detailed studies on miR159 are also needed to explore the potential in the improvement of drought tolerance.

## 5 Conclusion

In conclusion, the drought-associated miRNome of maize were analyzed by examining the drought-tolerant and drought-sensitive inbred lines grown in the field by deep sequencing. A total of 53 and 52 drought responsive miRNAs were identified in leaves and roots, respectively. Particularly, several miRNA-mRNA target modules showed uniquely responsive to drought in the two lines with contrasting drought tolerance. The miR164-MYB and miR164-NAC modules, regulated by ABA, were drought responsive specifically in H082183. The miR156-SPL module was uniquely responsive to drought in Lv28, with opposite regulation patterns in leaves and roots. In addition, our study detected three novel miRNAs with high confidence, which merit further validation and biological function studies. This study expands our knowledge of drought-induced molecular regulatory mechanisms modulated by miRNAs in maize and extends the list of candidate genes for molecular breeding for drought tolerance.

## Supporting information

S1 File Additional File (.zip) included Figs A-J and Tables A-J. **Fig A. Percentage of expressed miRNAs in each family.** (A) The expressed miRNAs of different families in leaves. (B) The expressed miRNAs of different families in roots. **Fig B. Distribution of length of known miRNAs that expressed in the present study. Fig C. The conservation analysis of predicted novel miRNAs.** The mature sequence of predicted novel miRNAs in the present study and known miRNAs in plants were aligned. (A) Novel_1_44595. (B) Novel_1_47179. (C) Novel_3_27520. (D) Novel_3_29234. (E) Novel_9_5030. (F) Novel_1-_3438. zma: *Zea mays*. osa: *Oryza sativa*. bdi: *Brachypodium distachyon*. tae: *Triticum aestivum*. mtr: *Medicago truncatula*. ath: *Arabidopsis thalian*a. ptc: *Populus trichocarp*a. vvi: *Vitis vinifera*. rco: *Ricinus communis*. **Fig D. The phylogenetic tree and sequence of three novel miRNAs.** The phylogenetic trees of Novel-2-38 (A), Novel-10-88 (B) and Novel-10-147 (C) were shown. The sequence of relative miRNAs in different species of Novel-2-38 (D), Novel-10-88 (E) and Novel-10-147 (F). ath: *Arabidopsis thaliana*, osa: *Oryza sativa*, sbi: *Sorghum bicolor*, zma: *Zea mays*. (G) The novel mature sequence of miRNAs were cloned using stem-loop methods in B73 (B), H082183 (H) and Lv28 (L). The maker (M) was 100-bp. **Fig E. Correlation result of miRNome in each replicate.** The TPM value of all known miRNAs in each replicate was calculated as log_10_(TPM+1) for the correlation analysis. The correlation coefficient r and the *P* value are labelled in each replicate sample. H and L indicate the two maize inbred lines H082183 and Lv28, respectively. MD and SD indicate moderate and severe drought, respectively. MC and SC indicate well-watered controls of moderate and severe drought, respectively. 1 and 2 indicate the two replicates. **Fig F. The T-plot of drought responsive miRNAs and their target genes.** (A) GRMZM2G065451, (B) GRMZM2G113779, (C) GRMZM2G414805, (D) GRMZM2G371033, (E) AC207656.3_FGT002, (F) GRMZM2G390641, (G) GRMZM2G159399, (H) GRMZM2G096358, (I) GRMZM2G114850, (J) GRMZM2G113779, (K) GRMZM5G806833, (L) GRMZM2G061734, (M) GRMZM2G004090, (N) GRMZM2G028054, (O) GRMZM2G304745, (P) GRMZM2G103812. **Fig G. The cluster analysis of genes.** (A) GRMZM2G096358 (MYB) and GRMZM2G114850 (NAC) in leaves of H082183. (B) GRMZM2G390641 (ARF) in leaves of Lv28. (C) GRMZM2G159399 (ARF) in leaves of Lv28. (D) AC207656.3_FG002 (ARF), GRMZM2G065451 (SPL) and GRMZM2G113779 (SPL) in leaves of Lv28. (E) GRMZM2G067624 (SPL) and GRMZM2G371033 (SPL) in leaves of Lv28. (F) GRMZM2G004090 (MYB) in roots of H082183. (G) GRMZM2G028054 (MYB) in roots of H082183. (H) GRMZM2G061734 (SPL) and GRMZM2G160917 (SPL) in roots of Lv28. (I) GRMZM2G460544 (SPL) and GRMZM5G806833 (SPL) in roots of Lv28. **Fig H. Co-expressed genes of miR156 targeted MYB and NAC genes that had over-presented transcription factor binding sequence in promoter regions.** The co-expressed genes of NAC and MYB genes that had significant over-presented MYB (A) or NAC (B) binding motif sequence in promoter regions were listed. The MYB (A) and NAC (B) binding sequences in promoter regions were plotted. **Fig I. The expression of miR159 and target genes in roots under ABA treatment.** (A) miR159. (B) GRMZM2G004090 (MYB). (C) GRMZM2G028054 (MYB). **Fig J. Correlation result of miRNA expression by qRT-PCR and sequencing.** Five miRNAs in leaves and five miRNAs in roots were selected to perform qRT-PCR validation under moderate drought and severe drought in H082183 and Lv28. **Table A. Primers used in the present study. Table B. Sequencing data of all 32 small RNA libraries.** ^a^ H: H082183; L: Lv28; MD: moderate drought; SD: severe drought; MC: well-watered control of moderate drought; SC: well-watered control of severe drought; 1 and 2: two replicates. **Table C. Result of reference genome alignment of small RNA reads.** ^a^ H: H082183; L: Lv28; MD: moderate drought; SD: severe drought; MC: well-watered control of moderate drought; SC: well-watered control of severe drought; 1 and 2: two replicates. **Table D. Alignment result of different small RNA databases.** ^a^ H: H082183; L: Lv28; MD: moderate drought; SD: severe drought; MC: well-watered control of moderate drought; SC: well-watered control of severe drought; 1 and 2: two replicates. **Table E. Numbers of expressed miRNAs in each sample.** ^a^ H: H082183; L: Lv28; MD: moderate drought; SD: severe drought; MC: well-watered control of moderate drought; SC: well-watered control of severe drought; 1 and 2: two replicates. **Table F. The data output of degradome sequencing. Table G. Predicted novel miRNAs.** ^a^ The number of libraries that had both miRNA and miRNA* reads. **Table H. Expression of all miRNA-mRNA pairs in leaves and roots. Table I. Drought responsive miRNAs in different species.** ^a^ miR156e-3p was down-regulated under moderate drought, while miR156b-5p and miR156d-5p were up-regulated under severe drought in Lv28. ^b^ miR164h-5p was down-regulated under moderate drought in H082183, but miR164c-3p and miR164h-3p were up-regulated under both moderate and severe drought in H082183. ^c^ miR171g-3p was down-regulated under moderate drought, but miR171h-3p and miR171k-3p were up-regulated under severe drought in Lv28. ^d^ miR156d-3p, miR156f-3p and miR156g-3p were down-regulated, while miR156e-3p was up-regulated under severe drought in H082183. ^e^ miR159a-3p, miR159b-3p, miR159f-3p, miR159j-3p and miR159k-3p were up-regulated under moderate drought in H082183, but miR159c-3p, miR159c-5p, miR159d-3p and miR159d-5p were down-regulated under moderate drought in Lv28. ^f^ miR167a-5p, miR167b-3p, miR167b-5p, miR167c-5p and miR167d-5p were up-regulated under severe drought in H082183, while miR167j-3p was down-regulated under moderate drought in Lv28. **Table J. Drought responsive miRNAs by using different drought treatment methods in maize.** ^a^ This line present the maize inbred lines used in experiment, in which 81565, 87–1, HKI-1532 and H082183 were drought tolerant lines, and 21ES, Dan340, V-372 and Lv28 were drought sensitive lines. ^b^ This line present the sampling time point after drought. The h and d present hours and days after drought treatment. ^c^ miR171h-3p and miR171k-3p were up-regulated, while miR171g-3p was down-regulated under drought. ^d^ miR156e-3p was up-regulated, while miR156d-3p, miR156f-3p and miR156g-3p were down-regulated under drought.
(ZIP)
