# Supplementary figures and images for: Genome-wide identification and comparative analysis of drought-related microRNAs in two maize inbred lines with contrasting drought tolerance by deep sequencing
Source: PLoS One. 2019 Jul 5;14(7):e0219176. doi: 10.1371/journal.pone.0219176 (PMC6611575; doi:10.1371/journal.pone.0219176)

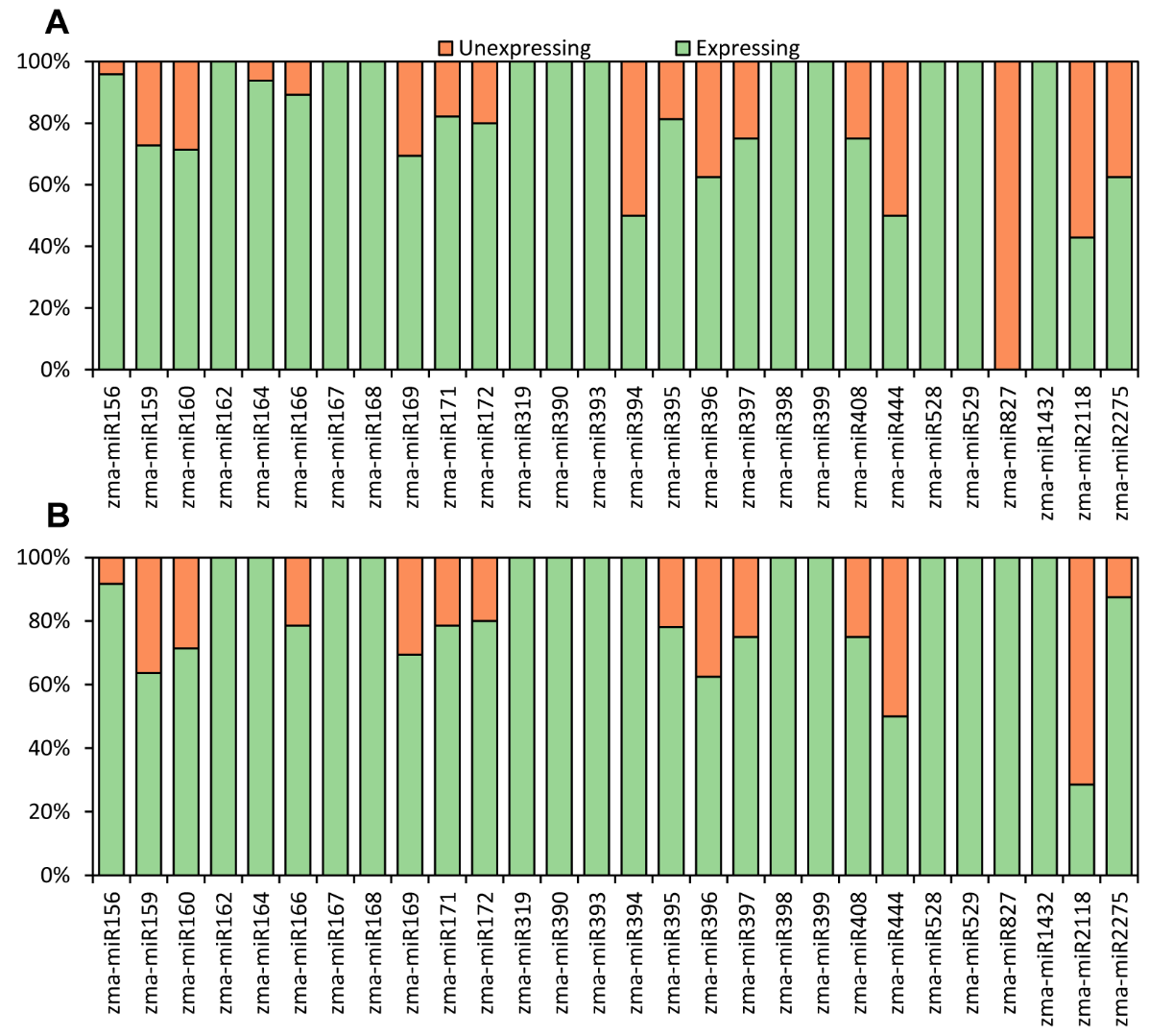

Supplement: S1 File — Additional File (.zip) included Figs A-J and Tables A-J. Fig A. Percentage of expressed miRNAs in each family. (A) The expressed miRNAs of different families in leaves. (B) The expressed miRNAs of different families in roots. Fig B. Distribution of length of known miRNAs that expressed in the present study. Fig C. The conservation analysis of predicted novel miRNAs. The mature sequence of predicted novel miRNAs in the present study and known miRNAs in plants were aligned. (A) Novel_1_44595. (B) Novel_1_47179. (C) Novel_3_27520. (D) Novel_3_29234. (E) Novel_9_5030. (F) Novel_1-_3438. zma: Zea mays. osa: Oryza sativa. bdi: Brachypodium distachyon. tae: Triticum aestivum. mtr: Medicago truncatula. ath: Arabidopsis thaliana. ptc: Populus trichocarpa. vvi: Vitis vinifera. rco: Ricinus communis. Fig D. The phylogenetic tree and sequence of three novel miRNAs. The phylogenetic trees of Novel-2-38 (A), Novel-10-88 (B) and Novel-10-147 (C) were shown. The sequence of relative miRNAs in different species of Novel-2-38 (D), Novel-10-88 (E) and Novel-10-147 (F). ath: Arabidopsis thaliana, osa: Oryza sativa, sbi: Sorghum bicolor, zma: Zea mays. (G) The novel mature sequence of miRNAs were cloned using stem-loop methods in B73 (B), H082183 (H) and Lv28 (L). The maker (M) was 100-bp. Fig E. Correlation result of miRNome in each replicate. The TPM value of all known miRNAs in each replicate was calculated as log10(TPM+1) for the correlation analysis. The correlation coefficient r and the P value are labelled in each replicate sample. H and L indicate the two maize inbred lines H082183 and Lv28, respectively. MD and SD indicate moderate and severe drought, respectively. MC and SC indicate well-watered controls of moderate and severe drought, respectively. 1 and 2 indicate the two replicates. Fig F. The T-plot of drought responsive miRNAs and their target genes. (A) GRMZM2G065451, (B) GRMZM2G113779, (C) GRMZM2G414805, (D) GRMZM2G371033, (E) AC207656.3_FGT002, (F) GRMZM2G390641, (G) G [file pone.0219176.s001.zip › Fig A.tif]

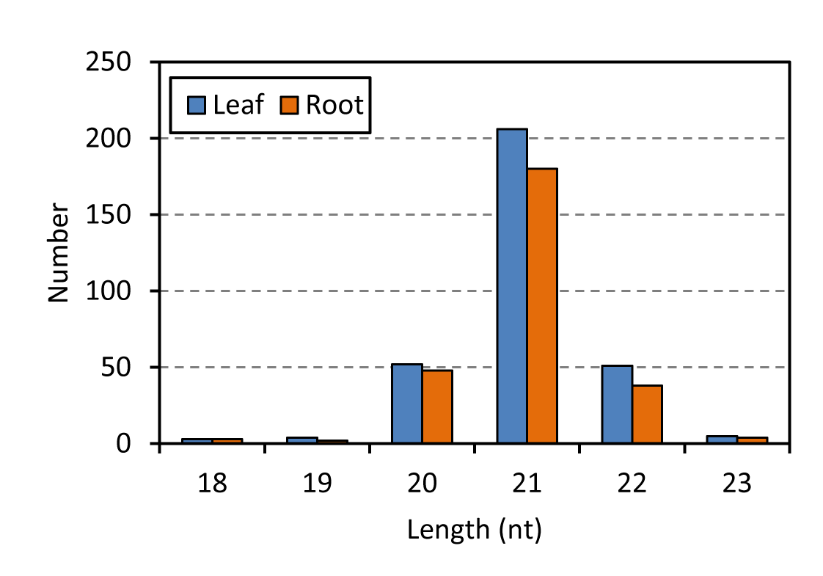

Supplement: S1 File — Additional File (.zip) included Figs A-J and Tables A-J. Fig A. Percentage of expressed miRNAs in each family. (A) The expressed miRNAs of different families in leaves. (B) The expressed miRNAs of different families in roots. Fig B. Distribution of length of known miRNAs that expressed in the present study. Fig C. The conservation analysis of predicted novel miRNAs. The mature sequence of predicted novel miRNAs in the present study and known miRNAs in plants were aligned. (A) Novel_1_44595. (B) Novel_1_47179. (C) Novel_3_27520. (D) Novel_3_29234. (E) Novel_9_5030. (F) Novel_1-_3438. zma: Zea mays. osa: Oryza sativa. bdi: Brachypodium distachyon. tae: Triticum aestivum. mtr: Medicago truncatula. ath: Arabidopsis thaliana. ptc: Populus trichocarpa. vvi: Vitis vinifera. rco: Ricinus communis. Fig D. The phylogenetic tree and sequence of three novel miRNAs. The phylogenetic trees of Novel-2-38 (A), Novel-10-88 (B) and Novel-10-147 (C) were shown. The sequence of relative miRNAs in different species of Novel-2-38 (D), Novel-10-88 (E) and Novel-10-147 (F). ath: Arabidopsis thaliana, osa: Oryza sativa, sbi: Sorghum bicolor, zma: Zea mays. (G) The novel mature sequence of miRNAs were cloned using stem-loop methods in B73 (B), H082183 (H) and Lv28 (L). The maker (M) was 100-bp. Fig E. Correlation result of miRNome in each replicate. The TPM value of all known miRNAs in each replicate was calculated as log10(TPM+1) for the correlation analysis. The correlation coefficient r and the P value are labelled in each replicate sample. H and L indicate the two maize inbred lines H082183 and Lv28, respectively. MD and SD indicate moderate and severe drought, respectively. MC and SC indicate well-watered controls of moderate and severe drought, respectively. 1 and 2 indicate the two replicates. Fig F. The T-plot of drought responsive miRNAs and their target genes. (A) GRMZM2G065451, (B) GRMZM2G113779, (C) GRMZM2G414805, (D) GRMZM2G371033, (E) AC207656.3_FGT002, (F) GRMZM2G390641, (G) G [file pone.0219176.s001.zip › Fig B.tif]

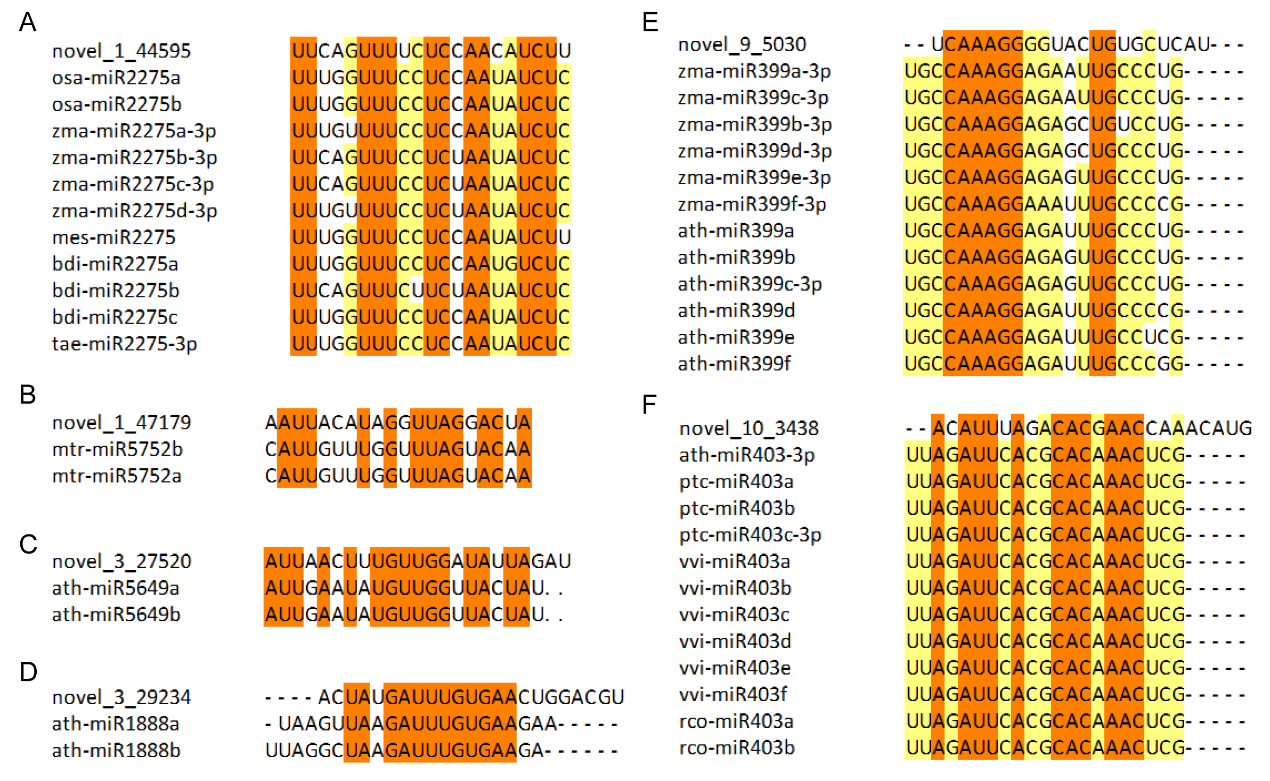

Supplement: S1 File — Additional File (.zip) included Figs A-J and Tables A-J. Fig A. Percentage of expressed miRNAs in each family. (A) The expressed miRNAs of different families in leaves. (B) The expressed miRNAs of different families in roots. Fig B. Distribution of length of known miRNAs that expressed in the present study. Fig C. The conservation analysis of predicted novel miRNAs. The mature sequence of predicted novel miRNAs in the present study and known miRNAs in plants were aligned. (A) Novel_1_44595. (B) Novel_1_47179. (C) Novel_3_27520. (D) Novel_3_29234. (E) Novel_9_5030. (F) Novel_1-_3438. zma: Zea mays. osa: Oryza sativa. bdi: Brachypodium distachyon. tae: Triticum aestivum. mtr: Medicago truncatula. ath: Arabidopsis thaliana. ptc: Populus trichocarpa. vvi: Vitis vinifera. rco: Ricinus communis. Fig D. The phylogenetic tree and sequence of three novel miRNAs. The phylogenetic trees of Novel-2-38 (A), Novel-10-88 (B) and Novel-10-147 (C) were shown. The sequence of relative miRNAs in different species of Novel-2-38 (D), Novel-10-88 (E) and Novel-10-147 (F). ath: Arabidopsis thaliana, osa: Oryza sativa, sbi: Sorghum bicolor, zma: Zea mays. (G) The novel mature sequence of miRNAs were cloned using stem-loop methods in B73 (B), H082183 (H) and Lv28 (L). The maker (M) was 100-bp. Fig E. Correlation result of miRNome in each replicate. The TPM value of all known miRNAs in each replicate was calculated as log10(TPM+1) for the correlation analysis. The correlation coefficient r and the P value are labelled in each replicate sample. H and L indicate the two maize inbred lines H082183 and Lv28, respectively. MD and SD indicate moderate and severe drought, respectively. MC and SC indicate well-watered controls of moderate and severe drought, respectively. 1 and 2 indicate the two replicates. Fig F. The T-plot of drought responsive miRNAs and their target genes. (A) GRMZM2G065451, (B) GRMZM2G113779, (C) GRMZM2G414805, (D) GRMZM2G371033, (E) AC207656.3_FGT002, (F) GRMZM2G390641, (G) G [file pone.0219176.s001.zip › Fig C.tif]

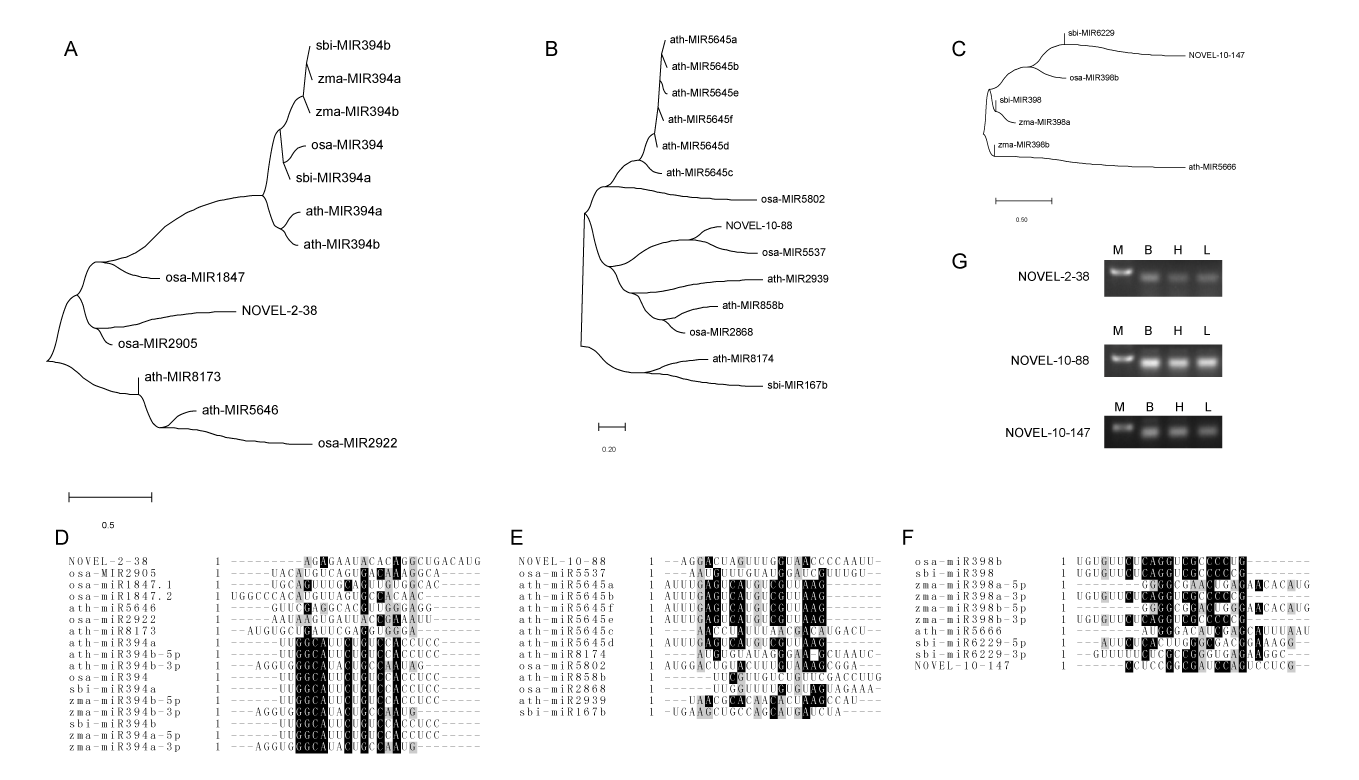

Supplement: S1 File — Additional File (.zip) included Figs A-J and Tables A-J. Fig A. Percentage of expressed miRNAs in each family. (A) The expressed miRNAs of different families in leaves. (B) The expressed miRNAs of different families in roots. Fig B. Distribution of length of known miRNAs that expressed in the present study. Fig C. The conservation analysis of predicted novel miRNAs. The mature sequence of predicted novel miRNAs in the present study and known miRNAs in plants were aligned. (A) Novel_1_44595. (B) Novel_1_47179. (C) Novel_3_27520. (D) Novel_3_29234. (E) Novel_9_5030. (F) Novel_1-_3438. zma: Zea mays. osa: Oryza sativa. bdi: Brachypodium distachyon. tae: Triticum aestivum. mtr: Medicago truncatula. ath: Arabidopsis thaliana. ptc: Populus trichocarpa. vvi: Vitis vinifera. rco: Ricinus communis. Fig D. The phylogenetic tree and sequence of three novel miRNAs. The phylogenetic trees of Novel-2-38 (A), Novel-10-88 (B) and Novel-10-147 (C) were shown. The sequence of relative miRNAs in different species of Novel-2-38 (D), Novel-10-88 (E) and Novel-10-147 (F). ath: Arabidopsis thaliana, osa: Oryza sativa, sbi: Sorghum bicolor, zma: Zea mays. (G) The novel mature sequence of miRNAs were cloned using stem-loop methods in B73 (B), H082183 (H) and Lv28 (L). The maker (M) was 100-bp. Fig E. Correlation result of miRNome in each replicate. The TPM value of all known miRNAs in each replicate was calculated as log10(TPM+1) for the correlation analysis. The correlation coefficient r and the P value are labelled in each replicate sample. H and L indicate the two maize inbred lines H082183 and Lv28, respectively. MD and SD indicate moderate and severe drought, respectively. MC and SC indicate well-watered controls of moderate and severe drought, respectively. 1 and 2 indicate the two replicates. Fig F. The T-plot of drought responsive miRNAs and their target genes. (A) GRMZM2G065451, (B) GRMZM2G113779, (C) GRMZM2G414805, (D) GRMZM2G371033, (E) AC207656.3_FGT002, (F) GRMZM2G390641, (G) G [file pone.0219176.s001.zip › Fig D.tif]

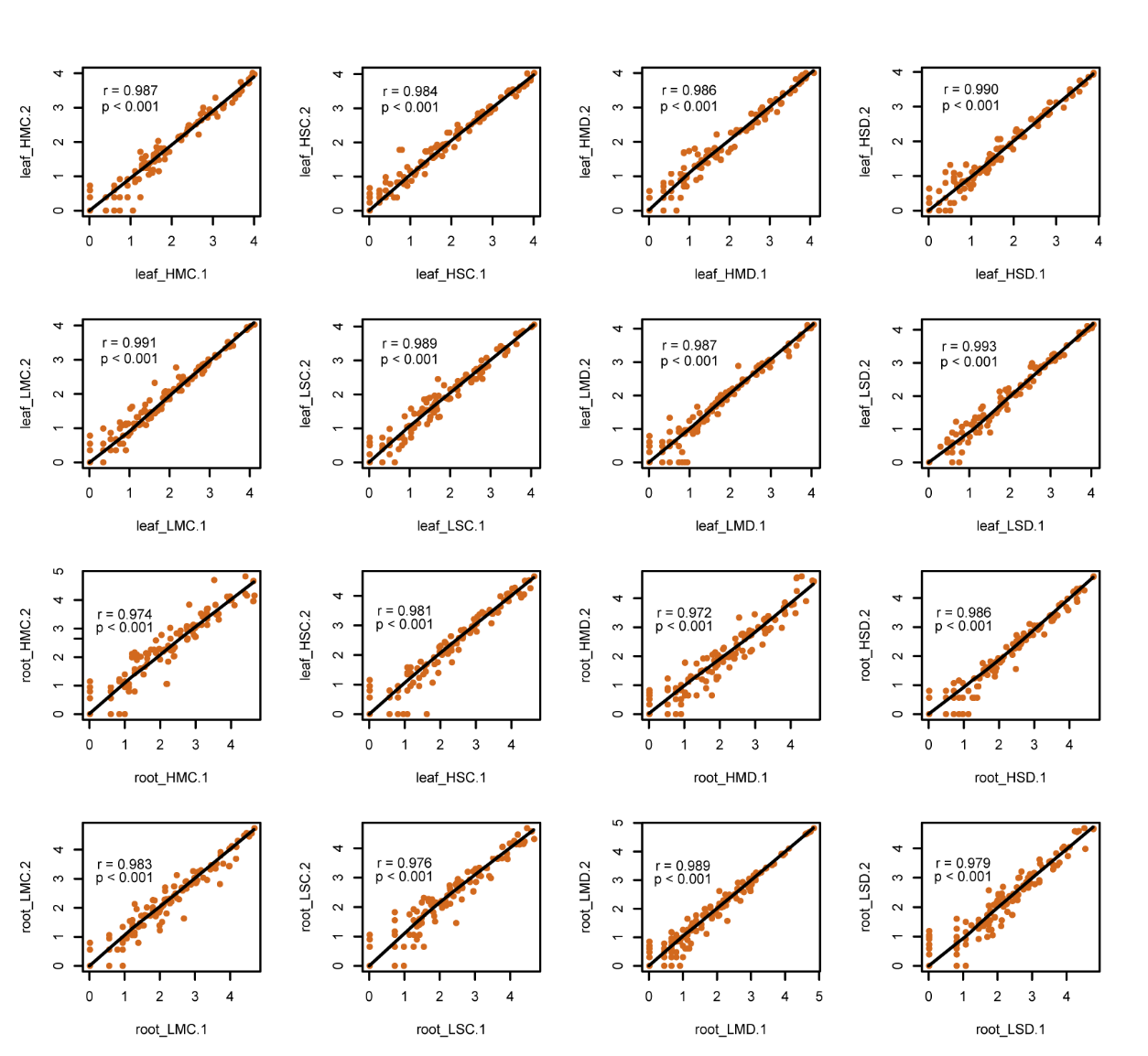

Supplement: S1 File — Additional File (.zip) included Figs A-J and Tables A-J. Fig A. Percentage of expressed miRNAs in each family. (A) The expressed miRNAs of different families in leaves. (B) The expressed miRNAs of different families in roots. Fig B. Distribution of length of known miRNAs that expressed in the present study. Fig C. The conservation analysis of predicted novel miRNAs. The mature sequence of predicted novel miRNAs in the present study and known miRNAs in plants were aligned. (A) Novel_1_44595. (B) Novel_1_47179. (C) Novel_3_27520. (D) Novel_3_29234. (E) Novel_9_5030. (F) Novel_1-_3438. zma: Zea mays. osa: Oryza sativa. bdi: Brachypodium distachyon. tae: Triticum aestivum. mtr: Medicago truncatula. ath: Arabidopsis thaliana. ptc: Populus trichocarpa. vvi: Vitis vinifera. rco: Ricinus communis. Fig D. The phylogenetic tree and sequence of three novel miRNAs. The phylogenetic trees of Novel-2-38 (A), Novel-10-88 (B) and Novel-10-147 (C) were shown. The sequence of relative miRNAs in different species of Novel-2-38 (D), Novel-10-88 (E) and Novel-10-147 (F). ath: Arabidopsis thaliana, osa: Oryza sativa, sbi: Sorghum bicolor, zma: Zea mays. (G) The novel mature sequence of miRNAs were cloned using stem-loop methods in B73 (B), H082183 (H) and Lv28 (L). The maker (M) was 100-bp. Fig E. Correlation result of miRNome in each replicate. The TPM value of all known miRNAs in each replicate was calculated as log10(TPM+1) for the correlation analysis. The correlation coefficient r and the P value are labelled in each replicate sample. H and L indicate the two maize inbred lines H082183 and Lv28, respectively. MD and SD indicate moderate and severe drought, respectively. MC and SC indicate well-watered controls of moderate and severe drought, respectively. 1 and 2 indicate the two replicates. Fig F. The T-plot of drought responsive miRNAs and their target genes. (A) GRMZM2G065451, (B) GRMZM2G113779, (C) GRMZM2G414805, (D) GRMZM2G371033, (E) AC207656.3_FGT002, (F) GRMZM2G390641, (G) G [file pone.0219176.s001.zip › Fig E.tif]

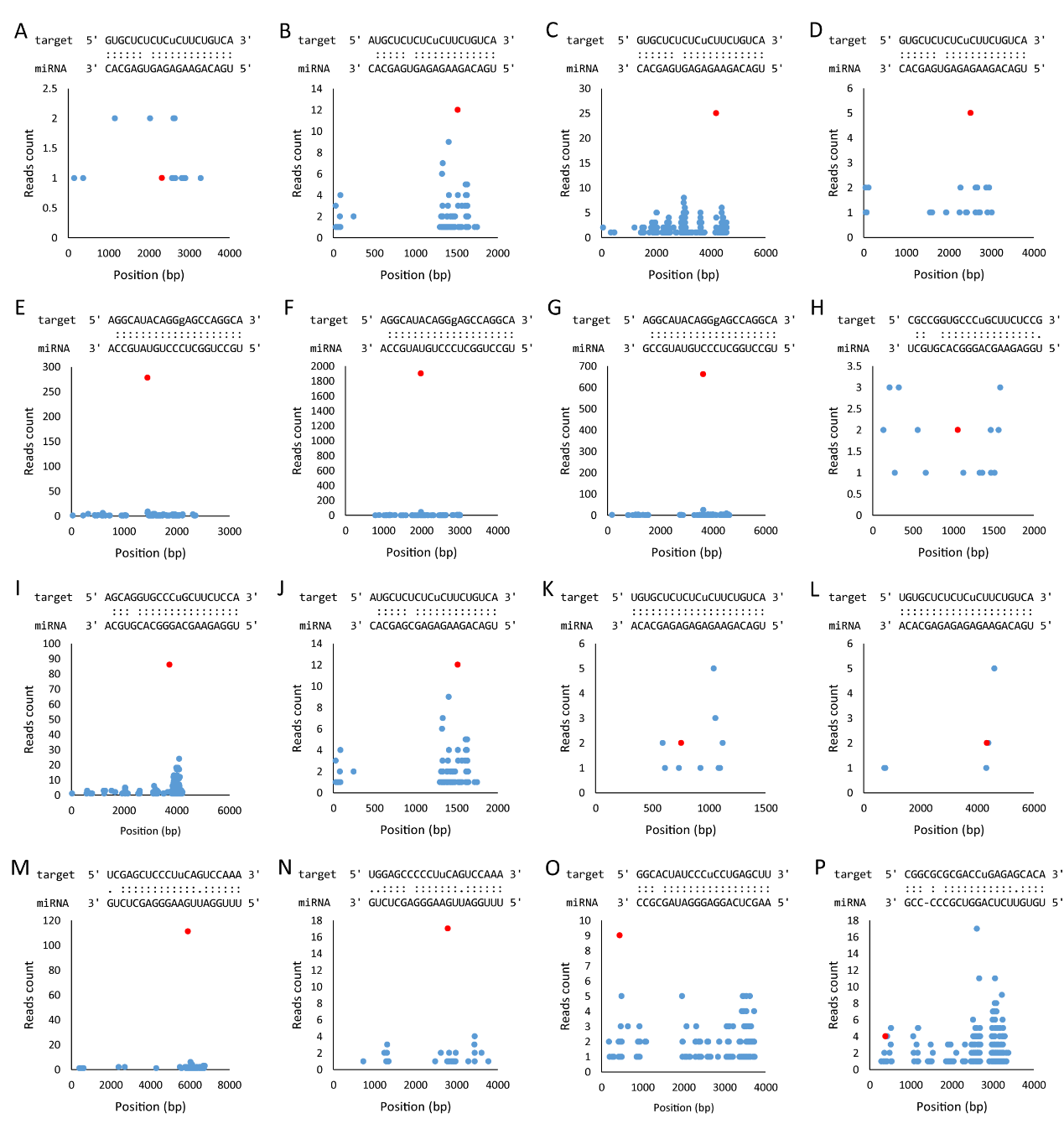

Supplement: S1 File — Additional File (.zip) included Figs A-J and Tables A-J. Fig A. Percentage of expressed miRNAs in each family. (A) The expressed miRNAs of different families in leaves. (B) The expressed miRNAs of different families in roots. Fig B. Distribution of length of known miRNAs that expressed in the present study. Fig C. The conservation analysis of predicted novel miRNAs. The mature sequence of predicted novel miRNAs in the present study and known miRNAs in plants were aligned. (A) Novel_1_44595. (B) Novel_1_47179. (C) Novel_3_27520. (D) Novel_3_29234. (E) Novel_9_5030. (F) Novel_1-_3438. zma: Zea mays. osa: Oryza sativa. bdi: Brachypodium distachyon. tae: Triticum aestivum. mtr: Medicago truncatula. ath: Arabidopsis thaliana. ptc: Populus trichocarpa. vvi: Vitis vinifera. rco: Ricinus communis. Fig D. The phylogenetic tree and sequence of three novel miRNAs. The phylogenetic trees of Novel-2-38 (A), Novel-10-88 (B) and Novel-10-147 (C) were shown. The sequence of relative miRNAs in different species of Novel-2-38 (D), Novel-10-88 (E) and Novel-10-147 (F). ath: Arabidopsis thaliana, osa: Oryza sativa, sbi: Sorghum bicolor, zma: Zea mays. (G) The novel mature sequence of miRNAs were cloned using stem-loop methods in B73 (B), H082183 (H) and Lv28 (L). The maker (M) was 100-bp. Fig E. Correlation result of miRNome in each replicate. The TPM value of all known miRNAs in each replicate was calculated as log10(TPM+1) for the correlation analysis. The correlation coefficient r and the P value are labelled in each replicate sample. H and L indicate the two maize inbred lines H082183 and Lv28, respectively. MD and SD indicate moderate and severe drought, respectively. MC and SC indicate well-watered controls of moderate and severe drought, respectively. 1 and 2 indicate the two replicates. Fig F. The T-plot of drought responsive miRNAs and their target genes. (A) GRMZM2G065451, (B) GRMZM2G113779, (C) GRMZM2G414805, (D) GRMZM2G371033, (E) AC207656.3_FGT002, (F) GRMZM2G390641, (G) G [file pone.0219176.s001.zip › Fig F.tif]

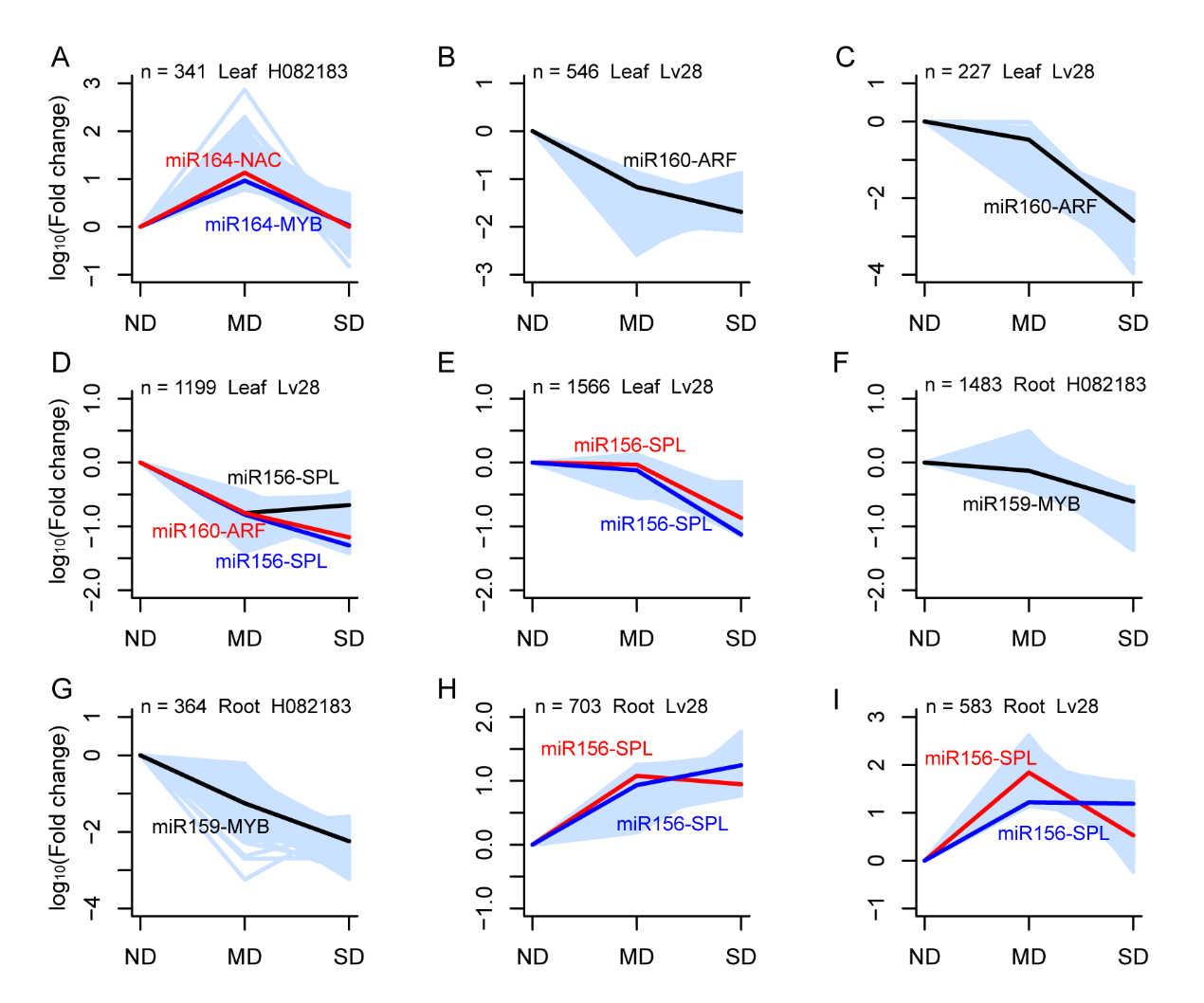

Supplement: S1 File — Additional File (.zip) included Figs A-J and Tables A-J. Fig A. Percentage of expressed miRNAs in each family. (A) The expressed miRNAs of different families in leaves. (B) The expressed miRNAs of different families in roots. Fig B. Distribution of length of known miRNAs that expressed in the present study. Fig C. The conservation analysis of predicted novel miRNAs. The mature sequence of predicted novel miRNAs in the present study and known miRNAs in plants were aligned. (A) Novel_1_44595. (B) Novel_1_47179. (C) Novel_3_27520. (D) Novel_3_29234. (E) Novel_9_5030. (F) Novel_1-_3438. zma: Zea mays. osa: Oryza sativa. bdi: Brachypodium distachyon. tae: Triticum aestivum. mtr: Medicago truncatula. ath: Arabidopsis thaliana. ptc: Populus trichocarpa. vvi: Vitis vinifera. rco: Ricinus communis. Fig D. The phylogenetic tree and sequence of three novel miRNAs. The phylogenetic trees of Novel-2-38 (A), Novel-10-88 (B) and Novel-10-147 (C) were shown. The sequence of relative miRNAs in different species of Novel-2-38 (D), Novel-10-88 (E) and Novel-10-147 (F). ath: Arabidopsis thaliana, osa: Oryza sativa, sbi: Sorghum bicolor, zma: Zea mays. (G) The novel mature sequence of miRNAs were cloned using stem-loop methods in B73 (B), H082183 (H) and Lv28 (L). The maker (M) was 100-bp. Fig E. Correlation result of miRNome in each replicate. The TPM value of all known miRNAs in each replicate was calculated as log10(TPM+1) for the correlation analysis. The correlation coefficient r and the P value are labelled in each replicate sample. H and L indicate the two maize inbred lines H082183 and Lv28, respectively. MD and SD indicate moderate and severe drought, respectively. MC and SC indicate well-watered controls of moderate and severe drought, respectively. 1 and 2 indicate the two replicates. Fig F. The T-plot of drought responsive miRNAs and their target genes. (A) GRMZM2G065451, (B) GRMZM2G113779, (C) GRMZM2G414805, (D) GRMZM2G371033, (E) AC207656.3_FGT002, (F) GRMZM2G390641, (G) G [file pone.0219176.s001.zip › Fig G.tif]

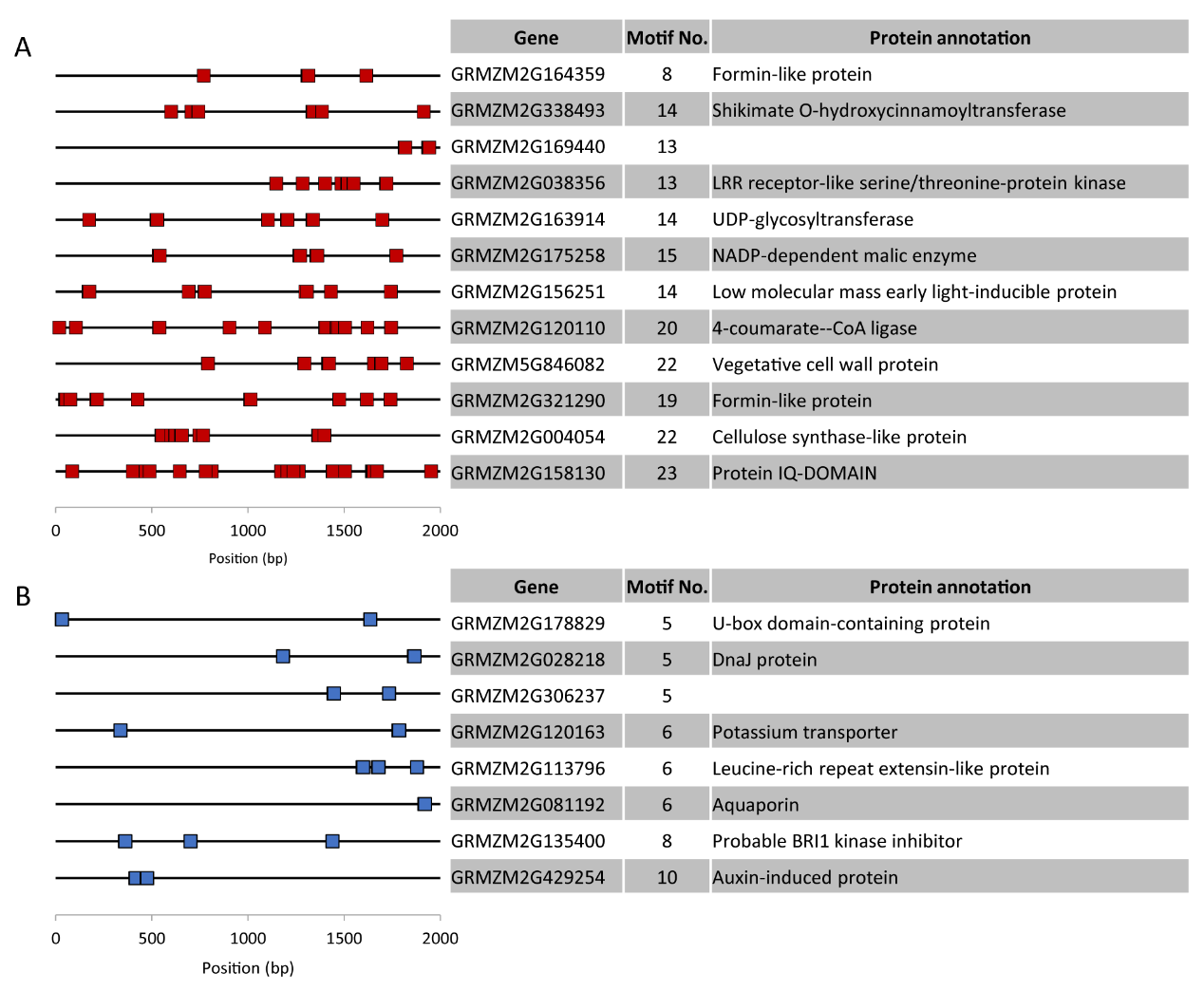

Supplement: S1 File — Additional File (.zip) included Figs A-J and Tables A-J. Fig A. Percentage of expressed miRNAs in each family. (A) The expressed miRNAs of different families in leaves. (B) The expressed miRNAs of different families in roots. Fig B. Distribution of length of known miRNAs that expressed in the present study. Fig C. The conservation analysis of predicted novel miRNAs. The mature sequence of predicted novel miRNAs in the present study and known miRNAs in plants were aligned. (A) Novel_1_44595. (B) Novel_1_47179. (C) Novel_3_27520. (D) Novel_3_29234. (E) Novel_9_5030. (F) Novel_1-_3438. zma: Zea mays. osa: Oryza sativa. bdi: Brachypodium distachyon. tae: Triticum aestivum. mtr: Medicago truncatula. ath: Arabidopsis thaliana. ptc: Populus trichocarpa. vvi: Vitis vinifera. rco: Ricinus communis. Fig D. The phylogenetic tree and sequence of three novel miRNAs. The phylogenetic trees of Novel-2-38 (A), Novel-10-88 (B) and Novel-10-147 (C) were shown. The sequence of relative miRNAs in different species of Novel-2-38 (D), Novel-10-88 (E) and Novel-10-147 (F). ath: Arabidopsis thaliana, osa: Oryza sativa, sbi: Sorghum bicolor, zma: Zea mays. (G) The novel mature sequence of miRNAs were cloned using stem-loop methods in B73 (B), H082183 (H) and Lv28 (L). The maker (M) was 100-bp. Fig E. Correlation result of miRNome in each replicate. The TPM value of all known miRNAs in each replicate was calculated as log10(TPM+1) for the correlation analysis. The correlation coefficient r and the P value are labelled in each replicate sample. H and L indicate the two maize inbred lines H082183 and Lv28, respectively. MD and SD indicate moderate and severe drought, respectively. MC and SC indicate well-watered controls of moderate and severe drought, respectively. 1 and 2 indicate the two replicates. Fig F. The T-plot of drought responsive miRNAs and their target genes. (A) GRMZM2G065451, (B) GRMZM2G113779, (C) GRMZM2G414805, (D) GRMZM2G371033, (E) AC207656.3_FGT002, (F) GRMZM2G390641, (G) G [file pone.0219176.s001.zip › Fig H.tif]

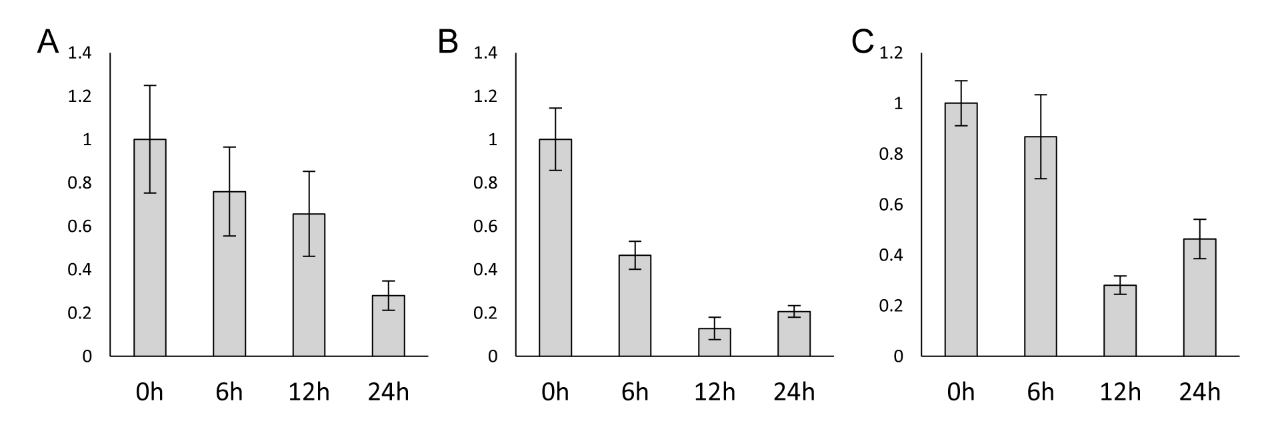

Supplement: S1 File — Additional File (.zip) included Figs A-J and Tables A-J. Fig A. Percentage of expressed miRNAs in each family. (A) The expressed miRNAs of different families in leaves. (B) The expressed miRNAs of different families in roots. Fig B. Distribution of length of known miRNAs that expressed in the present study. Fig C. The conservation analysis of predicted novel miRNAs. The mature sequence of predicted novel miRNAs in the present study and known miRNAs in plants were aligned. (A) Novel_1_44595. (B) Novel_1_47179. (C) Novel_3_27520. (D) Novel_3_29234. (E) Novel_9_5030. (F) Novel_1-_3438. zma: Zea mays. osa: Oryza sativa. bdi: Brachypodium distachyon. tae: Triticum aestivum. mtr: Medicago truncatula. ath: Arabidopsis thaliana. ptc: Populus trichocarpa. vvi: Vitis vinifera. rco: Ricinus communis. Fig D. The phylogenetic tree and sequence of three novel miRNAs. The phylogenetic trees of Novel-2-38 (A), Novel-10-88 (B) and Novel-10-147 (C) were shown. The sequence of relative miRNAs in different species of Novel-2-38 (D), Novel-10-88 (E) and Novel-10-147 (F). ath: Arabidopsis thaliana, osa: Oryza sativa, sbi: Sorghum bicolor, zma: Zea mays. (G) The novel mature sequence of miRNAs were cloned using stem-loop methods in B73 (B), H082183 (H) and Lv28 (L). The maker (M) was 100-bp. Fig E. Correlation result of miRNome in each replicate. The TPM value of all known miRNAs in each replicate was calculated as log10(TPM+1) for the correlation analysis. The correlation coefficient r and the P value are labelled in each replicate sample. H and L indicate the two maize inbred lines H082183 and Lv28, respectively. MD and SD indicate moderate and severe drought, respectively. MC and SC indicate well-watered controls of moderate and severe drought, respectively. 1 and 2 indicate the two replicates. Fig F. The T-plot of drought responsive miRNAs and their target genes. (A) GRMZM2G065451, (B) GRMZM2G113779, (C) GRMZM2G414805, (D) GRMZM2G371033, (E) AC207656.3_FGT002, (F) GRMZM2G390641, (G) G [file pone.0219176.s001.zip › Fig I.tif]

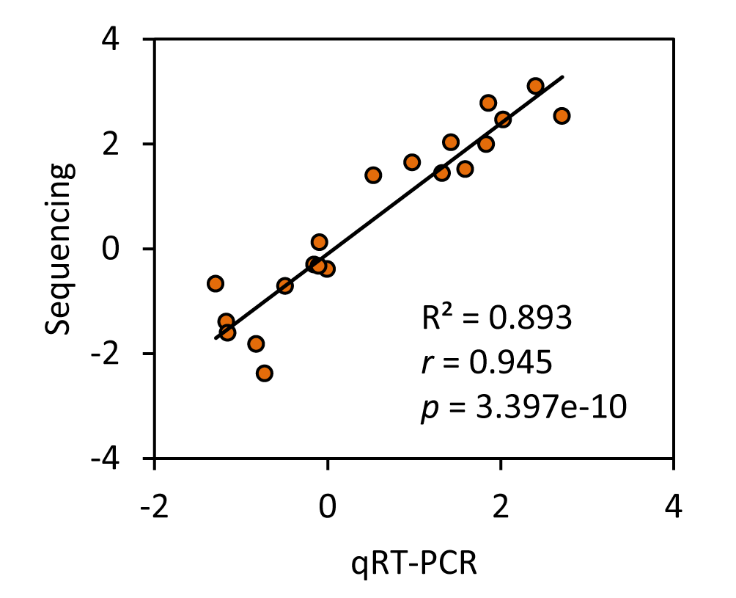

Supplement: S1 File — Additional File (.zip) included Figs A-J and Tables A-J. Fig A. Percentage of expressed miRNAs in each family. (A) The expressed miRNAs of different families in leaves. (B) The expressed miRNAs of different families in roots. Fig B. Distribution of length of known miRNAs that expressed in the present study. Fig C. The conservation analysis of predicted novel miRNAs. The mature sequence of predicted novel miRNAs in the present study and known miRNAs in plants were aligned. (A) Novel_1_44595. (B) Novel_1_47179. (C) Novel_3_27520. (D) Novel_3_29234. (E) Novel_9_5030. (F) Novel_1-_3438. zma: Zea mays. osa: Oryza sativa. bdi: Brachypodium distachyon. tae: Triticum aestivum. mtr: Medicago truncatula. ath: Arabidopsis thaliana. ptc: Populus trichocarpa. vvi: Vitis vinifera. rco: Ricinus communis. Fig D. The phylogenetic tree and sequence of three novel miRNAs. The phylogenetic trees of Novel-2-38 (A), Novel-10-88 (B) and Novel-10-147 (C) were shown. The sequence of relative miRNAs in different species of Novel-2-38 (D), Novel-10-88 (E) and Novel-10-147 (F). ath: Arabidopsis thaliana, osa: Oryza sativa, sbi: Sorghum bicolor, zma: Zea mays. (G) The novel mature sequence of miRNAs were cloned using stem-loop methods in B73 (B), H082183 (H) and Lv28 (L). The maker (M) was 100-bp. Fig E. Correlation result of miRNome in each replicate. The TPM value of all known miRNAs in each replicate was calculated as log10(TPM+1) for the correlation analysis. The correlation coefficient r and the P value are labelled in each replicate sample. H and L indicate the two maize inbred lines H082183 and Lv28, respectively. MD and SD indicate moderate and severe drought, respectively. MC and SC indicate well-watered controls of moderate and severe drought, respectively. 1 and 2 indicate the two replicates. Fig F. The T-plot of drought responsive miRNAs and their target genes. (A) GRMZM2G065451, (B) GRMZM2G113779, (C) GRMZM2G414805, (D) GRMZM2G371033, (E) AC207656.3_FGT002, (F) GRMZM2G390641, (G) G [file pone.0219176.s001.zip › Fig J.tif]
